# A multiscale model via single-cell transcriptomics reveals robust patterning mechanisms during early mammalian embryo development

**DOI:** 10.1371/journal.pcbi.1008571

**Published:** 2021-03-08

**Authors:** Zixuan Cang, Yangyang Wang, Qixuan Wang, Ken W. Y. Cho, William Holmes, Qing Nie

**Affiliations:** 1 Department of Mathematics, The NSF-Simons Center for Multiscale Cell Fate Research, University of California, Irvine, Irvine, California, United States of America; 2 Department of Mathematics, University of California, Riverside, Riverside, California, United States of America; 3 Department of Developmental and Cell Biology, University of California, Irvine, Irvine, California, United States of America; 4 Department of Physics and Astronomy, Department of Mathematics, Quantitative Systems Biology Center, Vanderbilt University, Nashville, Tennessee, United States of America; Purdue University, UNITED STATES

## Abstract

During early mammalian embryo development, a small number of cells make robust fate decisions at particular spatial locations in a tight time window to form inner cell mass (ICM), and later epiblast (Epi) and primitive endoderm (PE). While recent single-cell transcriptomics data allows scrutinization of heterogeneity of individual cells, consistent spatial and temporal mechanisms the early embryo utilize to robustly form the Epi/PE layers from ICM remain elusive. Here we build a multiscale three-dimensional model for mammalian embryo to recapitulate the observed patterning process from zygote to late blastocyst. By integrating the spatiotemporal information reconstructed from multiple single-cell transcriptomic datasets, the data-informed modeling analysis suggests two major processes critical to the formation of Epi/PE layers: a selective cell-cell adhesion mechanism (via EphA4/EphrinB2) for fate-location coordination and a temporal attenuation mechanism of cell signaling (via Fgf). Spatial imaging data and distinct subsets of single-cell gene expression data are then used to validate the predictions. Together, our study provides a multiscale framework that incorporates single-cell gene expression datasets to analyze gene regulations, cell-cell communications, and physical interactions among cells in complex geometries at single-cell resolution, with direct application to late-stage development of embryogenesis.

## Author summary

Starting as fertilized eggs, mammal embryos develop into fetuses with complex functions through robust spatiotemporal trajectotries. Correct timing of various regulatory mechanisms is an essential prerequisite that keeps developing biological systems on the right track. At the earliest stages of embryo development, cells make robust fate decisions to form inner cell mass which later develops into two cell types forming a particular spatial pattern. Through the lens of a multiscale three-dimensional model with the resolution of single cells in a realistic geometry, we study how timing of regulatory mechanisms ensures the robust developmental process in a tight time window. Assisted by single-cell transcriptomics data, the model revealed how the timing of a gene regulatory mechanism and a spatial mechanistic mechanism impact the pattern formation in early embryo development. We showed that both individual timings of these two mechanisms and the time overlap between them are essential to ensure correct pattern formation. We further validated our findings using distinct subsets of single-cell gene expression data and spatial imaging data. This data-informed multiscale modeling framework has a potential in studying other biological systems and developmental processes utilizing the emerging high-throughput and high-resolution data resources.

## Introduction

In mammals, the first two developmental events that occur are 1) the formation of the trophectoderm (TE) and inner cell mass (ICM) followed by 2) specification of the ICM into the primitive endoderm (PE) and epiblast (Epi). While both of these processes lead to the specification of primitive epithelial-like structures (the TE and PE) that wrap the future embryo (the Epi), the process that gives rise to the PE and TE are markedly different. While both are highly regulated processes, formation of the PE is both highly dynamic and stochastic by comparison. This raises the question, how can such a dynamic and stochastic process proceed robustly and reproducibly.

These first two developmental events lead to the formation of early multi-cellular structures that differ in both their gene expression and their location within the embryo. In the TE/ICM case, a monolayer shell of Cdx2 expressing TE cells surrounds an inner core of Oct4 expressing cells. In the Epi/PE case, an aggregate of Nanog expressing cells [1,2] forms the Epi, which is surrounded by PE, a monolayer of Gata6 expressing cells [3] that separates the Epi from embryonic cavity (blastocoel). These specification processes have a number of similarities. A tristable gene regulation circuit controls differentiation from an uncommitted state to one of two differentiated states in both cases [4–7]. Both also yield similar physical structures, an aggregate of cells surrounded by a monolayer.

Formation of the Epi and PE is however a distinctly more stochastic and dynamic process than TE/ICM formation. During formation of the TE/ICM, cells appear to choose their fate based on positional information (exterior cells become TE and interior cells become ICM). That is, cells differentiate in a mostly deterministic fashion and the TE and ICM structures are essentially constructed as a result of differentiation itself. While cells have been observed to move within the embryo, as few as 5% are exchanged between the TE and ICM [8]. Epi and PE cells on the other hand asynchronously (in time) differentiate to initially form a stochastically organized salt and pepper spatial distribution [9–12] that later evolves into the canonical Epi and PE structures through cellular motions.

Epi and PE differentiation is regulated by two mutually antagonistic factors, Nanog and Gata6 [1–3]. Prior to the 32-cell stage (~E3), these factors are co-expressed in almost all cells. By E3.5-E4, they are mutually exclusively expressed [11] in a salt and pepper distribution of Epi and PE cells. The proposed cause of this salt and pepper distribution is Fgf signaling [13–15], which is secreted by differentiated Epi cells and promotes expression of PE markers in neighbor cells. Interestingly, Epi/PE specification is not a bang-bang process at the population level. Instead, cells asynchronously differentiate at different times. While this could be viewed as a simple result of stochasticity, Saiz et al. [16] proposed the incremental commitment in conjunction with Fgf signaling is functionally important for controlling the proportions of PE and Epi cells. Numerical simulations verified this mechanism could robustly produce a salt and pepper distribution with proper cell proportions [13].

This still leaves the question of how these cells organize into canonical Epi and PE structures. Intercalation of cells into the PE layer due to blastocoel expansion contributes to PE formation [12]. Differential adhesion mediated sorting is also thought to play a crucial role [17,18]. While this idea sounds enticing however, adhesion factors that would facilitate this sorting have, to our knowledge, not been previously identified in the morula or early blastocyst stage mammalian embryos. Further, the presence of double positive (DP) cells expressing both Nanog and Gata6 has not previously been considered. Though these mechanisms have been investigated in isolated stages, a coherent understanding of how they corporate through multiple stages is still lacking.

A number of phenomenological modeling studies have been performed to study this developmental time frame. Non-spatial studies have helped identify the minimal gene interaction networks that regulate differentiation [4–6,19]. Spatial models have been used to study how mechanical factors such as cell-cell interactions [20] or cell contractility [21] influence development. Others have more comprehensively spatially modeled physical and regulatory processes [7,13,17,18]. Each of these studies have however been phenomenological in that they have largely integrated and been compared to imaging data and have not utilized the type of single cell RNA sequencing (scRNA-seq) data that has become available in recent years. Also, to the best of our knowledge, there have not been any three-dimensional models that comprehensively couple regulatory processes, spatial cell soring during Epi/PE separation, and single cell data to study Epi/PE formation.

Inspired by the promising results of data-centric approaches, several data-informed models have been introduced. For example, temporal models have incorporated either temporal RNA-sequencing data [22,23], or time series observations of neural activity [24] while spatiotemporal models have been calibrated with morphologic data [25]. These data-driven models depend on group average data without individual cell resolution. Recently the single-cell gene expression profiles become available for early mammalian embryos [26]. This opens up the opportunity of utilizing the data in modeling at a resolution of individual cells [27,28]. A single-cell qPCR dataset quantified 48 selected genes in mouse embryo from 1-cell stage to 64-cell stage [29]. Several recent scRNA-seq datasets on early mouse embryos provide an unbiased gene edxpression profiles of transcriptomics across different developmental stages, including E3.5-E6.5 [30], E5.25-E6.5 [31], and E6.5-E8.5 [32]. While, these scRNA-seq datasets allow us to explore the heterogeneity among individual cells, the spatial information is lost in scRNA-seq data, hindering the examination of communications among cells, which are crucial in cell fate decision. Further, currently no single dataset covering the time course from zygote to late blastocyst is available.

Here we develop a data-informed, three-dimensional multiscale model of mouse embryo development from the 1 to 128-cell stage to study how the dynamic interaction between the differentiation and sorting processes influences PE and Epi organization. This model couples 1) a model of gene regulation, 2) a family of models of adhesion mediated cell-cell interactions based directly on expression data for Eph/Ephrin pairs from single cell data, and 3) a 3D physical model of the embryo at a subcellular resolution. Using this combined approach, we uncovered that Eph/Ephrin ligand receptor pairings found in the single cell data provide the appropriate adhesion conditions to ensure the formation of PE and Epi. Furthermore, our simulations have revealed the importance of the duration of Fgf signaling during Epi and PE cell specification. While cell allocation and organization of Epi and PE cells is relatively insensitive to the timing of Fgf signaling onset, that signaling must be attenuated prior to the late 128-cell stage to ensure proper organization of embryo structures.

## Results

### A multiscale three-dimensional model from fertilization (1 cell) to late blastocyst (128 cells) stage

**Fig 1:**
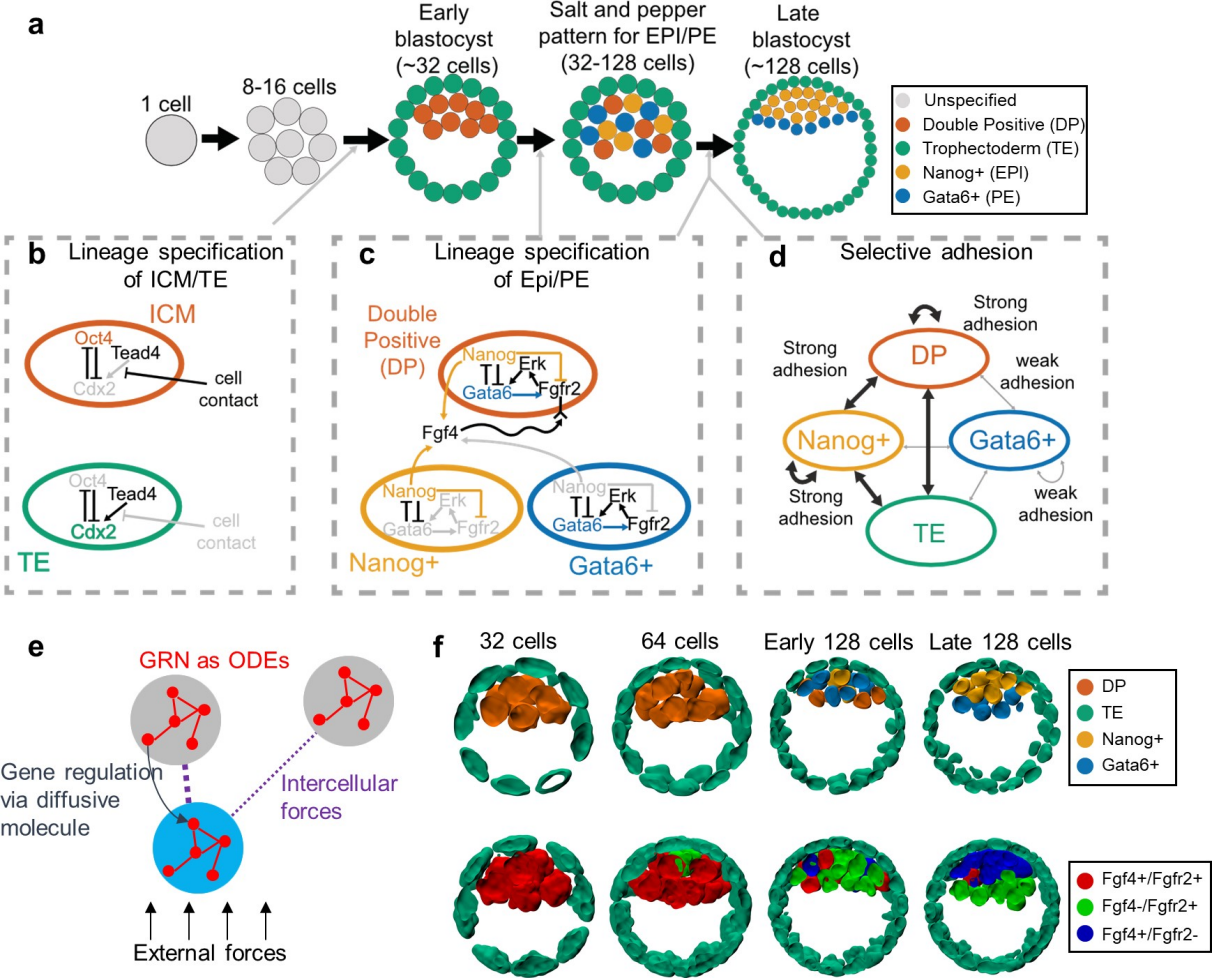
A multiscale model for early embryo development from fertilization to late blastocyst stage. (a) Embryo shapes in different stages. The circle color indicates the cell type: Grey for unspecified cell; Green for Trophectoderm (TE) cell; Red for inner Cell Mass: double positive of Nanog and Gata6 (DP); Yellow for Nanog high and Gata6 low (Nanog+); Blue for Gata6 high and Nanog low (Gata6+). (b) Gene regulation models for ICM specification before early blastocyst stage. The grey color represents weak cell contact or weak gene expression. (c) Gene regulation models for Nanog+/Gata6+ specification during early to late blastocyst stage. (d) Modeled selective adhesion between different cell types through early to late blastocyst stage. (e) A schematic illustration of the multiscale model incorporating spatial and gene expression dynamics of the cells. The correspondence to equations are as follows: GRNs, Eqs (1)–(5); intercellular forces, Eq (7); intercellular gene regulation, Eqs (5) and (6); external forces, S1 Text Eqs (3) and (4). (f) Simulated embryo with Nanog/Gata6 and Fgf4/Fgfr2 expression at different stages.

We constructed a 3D, multiscale spatial-temporal model for the development of the multicellular blastocyst from 1-cell to 128-cell stage (Fig 1A). The model couples two developmental processes that are critical to this early phase of development. 1) Regulation of cell fate specifying genes (Oct4/Cdx2 for TE/ICM and Nanog/Gata6 for Epi/PE) is modeled using ordinary differential equations (ODE) (Fig 1B and 1C). 2) Physical cell-cell interactions (including selective adhesion, Fig 1D) with subsequent cell migrations are modeled using the subcellular element method. This multiscale model (Fig 1E) recapitulates phenomenologically correct developmental process of the embryo from zygote to blastocyst (Fig 1F) [16,33]. We first briefly describe the gene regulatory model used and then subsequently describe the 3D modeling framework used to model the whole early embryo.

Gene regulatory dynamics associated with the TE/ICM and Epi/PE formations are modeled separately. Mutually antagonistic and self-activation dynamics of Oct4/Cdx2 modulated by cell contact (similar to [7]) are used to model TE/ICM formation (See S1 Text section 2 for detailed gene network equations). Similar mutually antagonistic dynamics between Nanog/Gata6 describe Epi/PE formation. In this case, cell-cell communication occurs via Fgf4/Fgfr2 regulation of the Erk signaling pathway (for simplicity, we refer to this as Fgf4/Fgfr2 or just Fgf signaling). The detailed equations are listed in Eqs (1)–(6).

To integrate these regulatory dynamics with the mechanical and morphological aspects of embryo development, we developed a 3D spatial model where the embryo is modeled by a collection of discretely represented cells constrained in a spherical geometry (inspired by [7,17]). The model was implemented in the framework of subcellular element method (SEM) [34] which represents a cell by a collection of elements (particles in 3D space). The spatial dynamics of the cells depicted by these elements partially depend on the modeled gene expression and, in turn, provide a spatial reference for modeling intercellular gene regulations [7,35,36]. The following aspects are accounted for: cell-cell interactions; selective adhesion; cell division; confinement of cells by the zona pellucida; and cavity formation (see Eq (7) and S1 Text section 1 for details).

The cell divisions are modeled by splitting the elements of a mother cell into two subsets representing the two daughter cells. The cell divisions are scheduled as follows: 1) from 1- to 32-cell stage, all cells divide at the same time, 2) from 32- to 64-cell stage and from 64- to 128-cell stage, the cell cycle for each cell is modeled by a random variable uniformly distributed over a time window.

Among the aspects modeled in the spatial model, we are especially interested in the consequences of selective adhesion to evaluate how heterogeneous cellular adhesion mechanism impacts the pattern formation in early embryo development. We represent the selective adhesion mechanisms by assigning adhesion scores (AS) for different cell type pairs. A high AS means a strong adhesion and a low AS means a weak adhesion. The AS is implemented as the parameter α in Eq (7). To quantify AS and model selective adhesion, we will take two approaches. First, we will use single-cell RNA sequencing data [30] to quantify expression levels of adhesion related molecules (Eqs (8) and (9)) and assign AS based on data. Second, we will explore the effectiveness of different phenomenological models of adhesion (encoded in the AS) to determine how different types of selective adhesion influence organization (see S1 Text section 3 for exact values used).

### Integrative data and model analysis reveals selective adhesion differences driven by EphA4/EphrinB2 heterogeneity promotes proper sorting of the PE and Epi

In the following results, we classify the inner cell mass during lineage specification as suggested in [16] into Nanog+ (cells expressing high Nanog and low Gata6 committed to Epi), Gata6+ (cells expressing high Gata6 and low Nanog committed to PE), double positive (DP, cells expressing high Nanog and Gata6) and double negative (DN, cells expressing low Nanog and Gata6). We call a simulated pattern successful if a single aggregation of Epi cells is formed and is attached to the TE shell covered by a PE layer [16,33]. A simulated pattern is classified as partial success if multiple Epi aggregates form but are still attached to the TE shell and covered by a PE layer. A simulated pattern is classified as failure if the embryo stays in salt-and-pepper pattern or Epi and PE form separate clusters. We have also developed a loss score to quantify the extent of divergence of a simulated pattern from the ideal pattern (see Materials and Methods: Embryo pattern loss score).

After initial cell fate specification, Nanog+ and Gata6+ cells form a salt and pepper configuration. Biased active cell movement mediated by intercellular interactions [12] has been suggested to lead to organization of the resulting PE and Epi structures (Fig 1A). Specifically, differential or selective adhesion has been proposed to sort cells of differing fates in a number of scenarios [37]. In the context of the embryo, prior phenomenological modeling demonstrated that selective adhesion between Epi/PE/TE can induce Epi/PE separation [17]. This study did not however account for the presence of DP cells (expressing both Nanog and Gata6), which were recently shown to co-exist with Nanog+ and Gata6+ cells [16] (Fig 1D). Further, candidate molecules that facilitate this selective adhesion have not been identified to our knowledge. We thus first investigate this sorting process accounting for the additional presence of DP cells and use single cell data to identify and test candidate adhesion molecules that may drive selective adhesion.

To incorporate a data-informed mechanism for cell sorting into the model, we first quantified expression levels of adhesion-repulsion associated genes for different cell types from single-cell RNA sequencing data [30]. A family of ligand-receptor pairs, Eph/Ephrin, has been shown to contribute to selective adhesion, and different pairs may lead to strengthened or weakened adhesion [38,39]. In particular, EphB2/EphrinB2 triggers repulsion [40], and EphA4/EphrinB2 increases adhesion [41]. Moreover, Eph/Ephrin pairs contribute to somite formation [42,43]. We thus use single-cell RNA-seq data on mouse early embryos (E3.5 to E6.75) to quantitatively assess expression levels of different Eph/Ephrin pairs [30].

**Fig 2:**
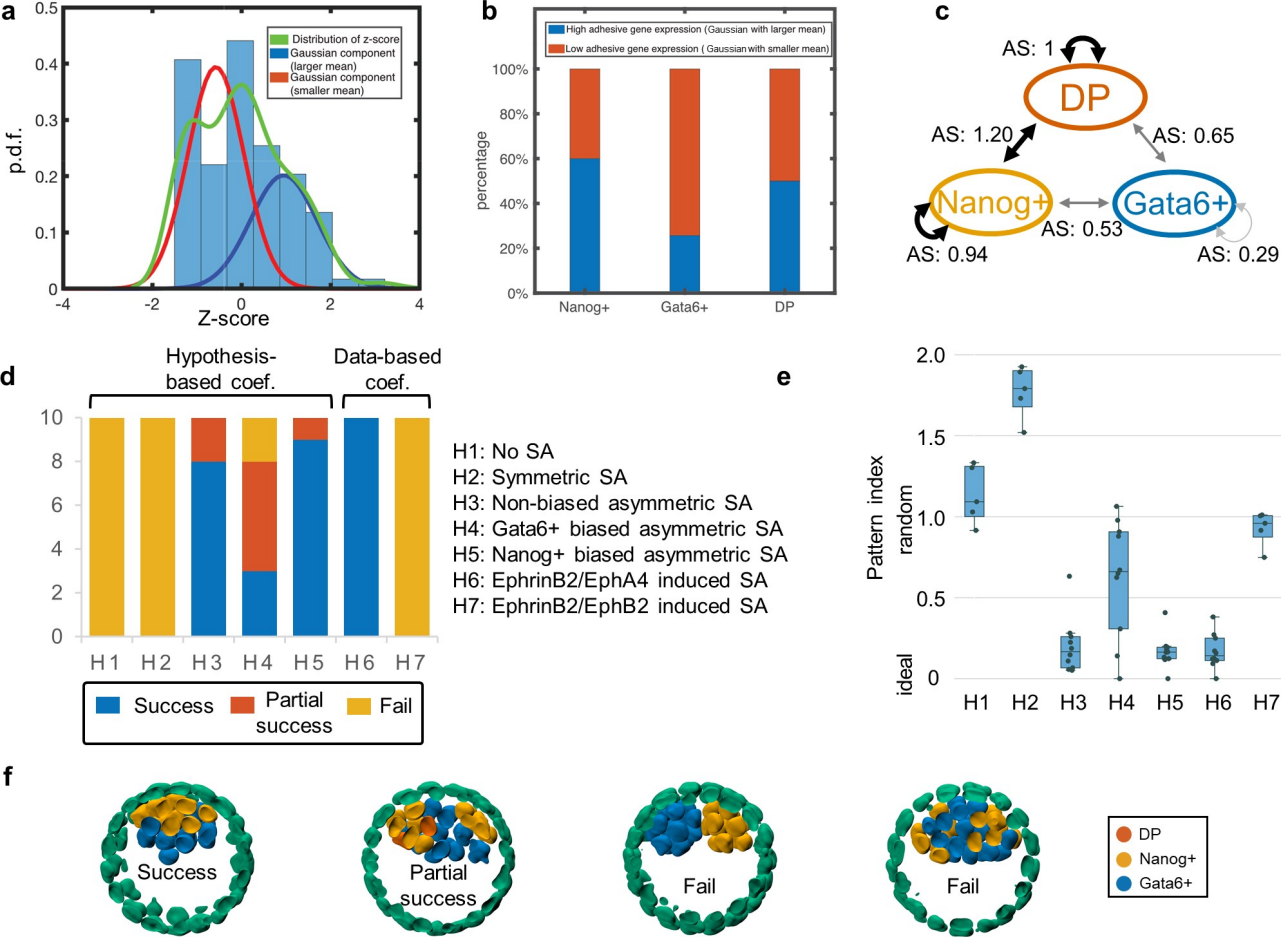
Data-informed selective adhesion model leads to successful cell arrangement at 128 cell stage. (a) Histogram of z-score of summation of log([EphA4] + 1) and log([EphrinB2] + 1) at E4.5 stage. The green curve shows the distribution of z-score. The red and blue curves show two components of Gaussian Mixture to fit the distribution. (b) Percentage of high/low adhesive gene expression levels in Nanog+/Gata6+/DP cells at E4.5 stage. (c) An EphA4/EphrinB2 driven selective adhesion mechanism between Nanog+/Gata6+/DP cells, where higher adhesion score (AS) indicates stronger adhesion, and a positive AS means strengthened adhesion. (d) Success rate for Nanog+/Gata6+ cells arrangement in simulations with different selective adhesion hypotheses: (H1) no selective adhesion; (H2) symmetric selective adhesion where adhesion between Nanog+/Nanog+, Gata6+/Gata6+ and DP/DP cells are the same; (H3) asymmetric selective adhesion where DP cells have same adhesion with Nanog+ and with Gata6+; (H4) asymmetric selective adhesion where DP cells have stronger adhesion with Nanog+ cells than with Gata6+ cells; (H5) asymmetric selective adhesion where DP cells have stronger adhesion with both Nanog+ and Gata6+ cells; (H6) the EphA4/EphrinB2 driven selective adhesion; (H7) the EphB2/EphrinB2 driven selective adhesion. (e) Pattern loss score of the simulations. Each data point corresponds to one simulation. A loss score of 0 indicates a perfect pattern and random cell type assignments have an expected loss score of 1. (f) Representative terminal Nanog+/Gata6+ cell arrangements for successful, partially successful, and failed cases.

We quantified the combined ligand/receptor expression level in the ICM using the logarithm of multiplication of ligand and receptor expression levels, based on the EphA4/EphrinB2 expression level in scRNA-seq data [30]. Quantification of these genes in the ICM shows that EphA4/EphrinB2 forms a two-mode Gaussian mixture distribution pattern (Fig 2A, green curve), which is a mixture of one with high adhesion gene expression (Fig 2A, blue curve) and one with low adhesion gene expression (red curve). Further analysis shows that different fractions of Nanog+/Gata6+/DP cells are in the high/low adhesion gene expression states (which is quantified through binarization based on the Gaussian mixture) (Fig 2B). Fewer Gata6+ cells highly express adhesion genes compared to Nanog+/DP cells, which suggests a stronger adhesion among Nanog+/DP cells than Gata6+ cells.

To test if this distribution of EphA4/EphrinB2 can drive correct spatial patterning, we first derived a data-informed selective adhesion model based on these expression levels. The selective adhesion is modeled by calculating adhesion scores (AS) directly from expression of ligand-receptor pairs in scRNA-seq data [29,44] as described in Eqs (8) and (9).

We focus primarily on the pair EphA4/EphrinB2 at E4.5. We also analyzed the EphB2/EphrinB2 combination (Fig 2D and 2E, model H7). However we are less confident in this data since EphB2 was unidentified in most cells, potentially due to dropout in the scRNA-seq data. Since our Eph/Ephrin quantification is limited to ICM cells due to the exclusion of TE cells in scRNA-seq data [30], we assumed adhesion between TE and Epi cells is stronger than that between PE and TE cells (following [17]). We also validated this mechanism (S1 Fig) by demonstrating that without this interaction, organization fails.

For simulations of this Eph/Ephrin based adhesion model, we allowed the embryo to develop *in silico* to the 64-cell stage and then turned on selective adhesion (further examination of the effects of this starting time are discussed in the next section). For EphA4/EphrinB2, the normalized adhesion strength (AS) is calculated using Eq (9) (Fig 2C). In the simulations, EphA4/EphrinB2 driven selective adhesion was able to generate correct spatial pattern (Fig 2D and 2E, model H6). See Fig 2F for examples of success, partial success and failure cases of pattern formation. Based on the limited data available, results suggest the EphB2/EphrinB2 model may be insufficient to achieve organization (though better single cell data is needed here). These simulation results suggest an EphA4/EphrinB2 driven model of selective adhesion is sufficient to organize the Epi/PE structures.

This is of course not the only possible model of selective adhesion. We thus further studied the space of potential selective adhesion possibilities using phenomenological adhesion models that simply assign cell type dependent adhesions. By studying a range of different cell-type dependent adhesion models (Figs 2 and S2), we identified two models that lead to effective organization (Fig 2D and 2E, models H3 and H5). In both effective models, there is a stronger adhesion among Nanog+ cells than that among Gata6+ cells and between Nanog+/Gata6+ cells. In model H5, DP cells have a stronger adhesion to Nanog+ cells whereas in H3, DP cells exhibit unbiased adhesion. Notably, model H5 recapitulates the qualitative dynamics predicted by the model using single cell expression of EphA4/EphrinB2.

In conclusion, the model suggests that EphA4/EphrinB2 distributions observed in scRNA data from the embryo are sufficient to promote Epi/PE sorting. The finding is consistent with the observatuion that EphA4 is upregulated in the inner cell mass of mouse and human embryos [45]. Further analysis of the space of possible selective adhesion models reveals two potential models that could in principle lead to proper organization. One of these two models has the exact qualitative structure found from the adhesion score analysis of EphA4/EphrinB2 interactions. Taken together, these results suggest this is a candidate ligand/receptor pair that drives sorting of the Epi/PE.

### Selective adhesion mechanism occurrence before 128-cell stage ensures correct Epi/PE pattern formation

**Fig 3:**
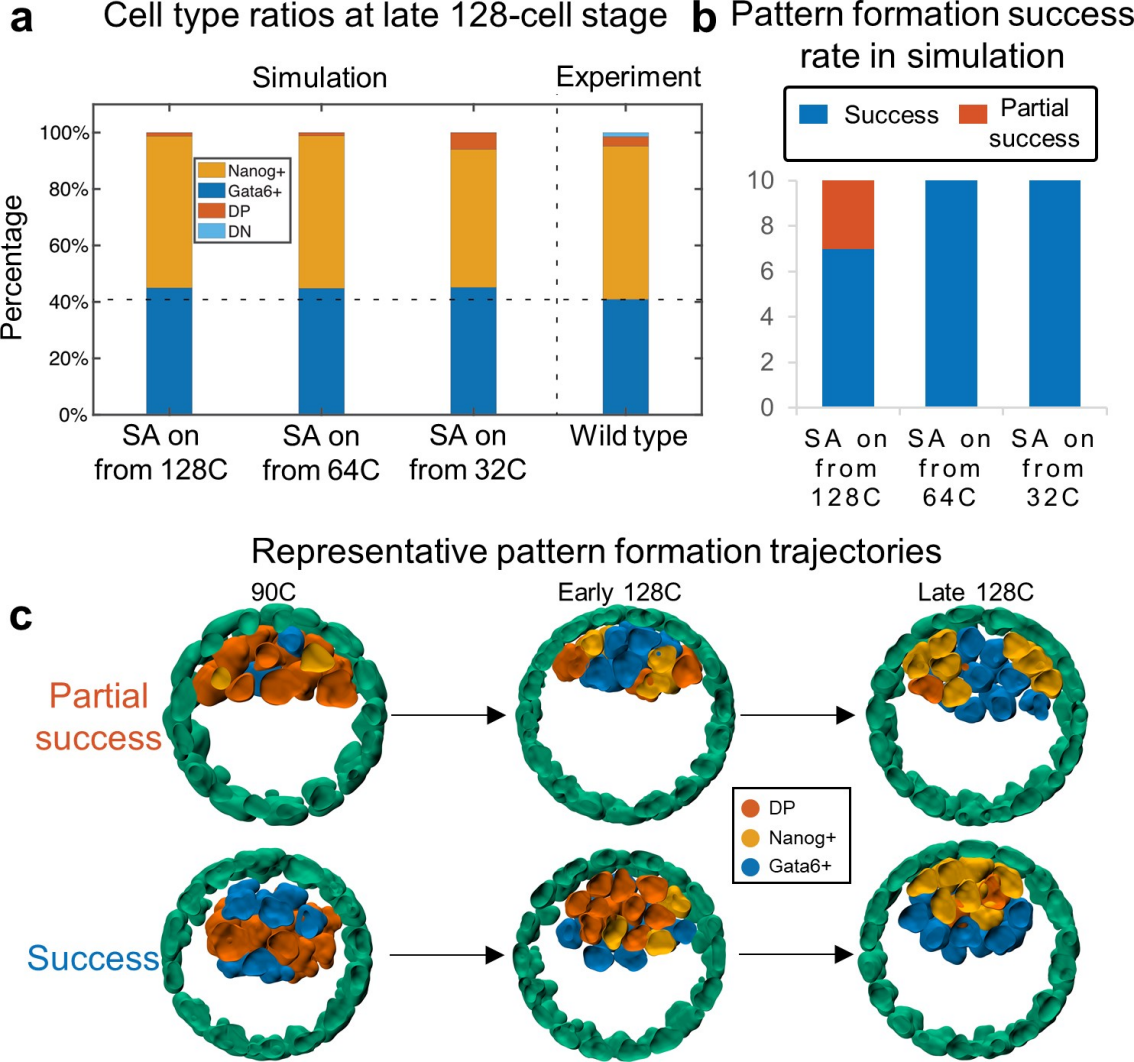
Sensitivity of selective adhesion starting time. (a) Ratio of Nanog+/Gata6+/DP/DN at 128-cell stage for different selective adhesion (SA) starting time; (b) Success rate for embryo development at 128-cell stage for different SA starting time. (c) Spatial pattern of simulation where selective adhesion starts from 128-cell stage (a partially successful case) and simulation where selective adhesion starts from 64-cell stage (a successful case).

We now consider the effect of timing of selective adhesion onset on embryonic organization. In the prior section, we artificially implemented this to occur at the 64-cell stage. It could however potentially take effect either earlier or later. Thus, we tested the EphA4/EphrinB2 driven mechanism with three different initiation times: immediately after the system reaches 32-cell stage, 64-cell stage or 128-cell stage. In the simulations, all three models give similar Nanog+/Gata6+/DP ratios round 40%/60%/0%, which are consistent with experimental data [16] (Fig 3A). These results suggest that the ratio between Nanog+/Gata6+/DP and spatial pattern are robust to the selective adhesion occurrence time. On the other hand, all the simulations with the selective adhesion starting time at 64- or 32-cell stage achieved correct final pattern (Fig 3B) while three out of ten simulations with the selective adhesion starting from 128-cell stage are only partially successful. In these partially successful cases, some Gata6+ cells aggregate near the TE (Fig 3C). This defect is potentially due to the absence of a time window overlap between the selective adhesion and the cell type transition from DP to Nanog+/Gata6+. These results suggest that while the Nanog+/Gata6+/DP ratio is robust to selective adhesion occurrence time, the corporation between selection adhesion and the cell fate regulation dynamics is crucial to the formation of correct spatial pattern.

We also evaluated the impact of the level of random cell movement (implemented as a Gaussian noise on subcellular element movement) on spatial pattern formation which is mainly driven by directed cell movements. Simulations were carried out with lower random cell movement level (1/10 of the amount of Gaussian noise compared to baseline simulation) and higher random cell movement level (5 times the amount of Gaussian noise compared to baseline simulation). Both Nanog/Gata6 expression levels and population ratio of Nanog+/Gata6+/DP were similar between these simulations and the baseline model (S3 Fig). With lower movement randomness, the inner cell mass had a flatter shape, and there were some misplaced Gata6+ cells, whereas, with higher movement randomness, the inner cell mass has a rounder shape with few misplaced cells. In summary, the population ratio between Nanog+/Gata6+/DP is relatively robust to the level of random movement but sufficiently large randomness of motion is required to reduce the instances of misplaced cells and ensure proper organization.

### Attenuation of Fgf signaling after Epi/PE formation is required to maintain organization

Nanog and Gata6 are key specification factors for Epi/PE cells [46]. Image data shows that Nanog/Gata6 expression levels are both high at early blastocyst (~32-cell stage) and become mutually exclusively expressed in Epi/PE cells at late blastocyst (~128-cell stage) with Nanog+/Gata6+ cells maintaining a relatively stable ratio in late blastocyst stage: 55%-60% for Gata6+ cell and 40%-45% for Nanog+ cell [16]. Experimental evidence suggests this dynamics of Nanog/Gata6 expression is regulated by Fgf4/Fgfr2 signaling. Nanog+ cells secrete Fgf4 signal. Activation of Fgfr2 receptors via Fgf4 binding promotes the expression of Gata6 and antagonizes Nanog expression [46]. A mathematical model with this Fgf/Fgfr/Erk signaling modulating Nanog/Gata6 expression was shown to generate appropriate fractions of Nanog+ and Gata6+ cells from the initial DP pool [19]. However, cell division and cell movement were excluded from this model, which are key processes in embryo development. We incorporated Fgf/Fgfr2/Erk signaling into a spatial model (including cell division and cell movement) to evaluate the role of Fgf signaling on Nanog+/Gata6+ specification and maintenance during embryo development.

First, we evaluated whether Fgf signaling regulation on Nanog/Gata6 is necessary for Nanog+/Gata6+ lineage specification. Simulations with only Nanog/Gata6 mutual cross inhibition but without Fgf signaling cannot lead to the cell fate separation into Nanog+ and Gata6+. These simulations lead to incorrect Nanog/Gata6 expression patterns (DN dominated, DP dominated, Nanog+ dominated), regardless of the basal expression rate or their cross inhibition strength (S4 Fig). Thus, as suggested by others, Fgf signaling regulation of Nanog/Gata6 likely ensures a correct Nanog+/Gata6+ population ratio. However, it is important to recall that the Fgf mediated specification process is occurring coincident with cell sorting.

**Fig 4:**
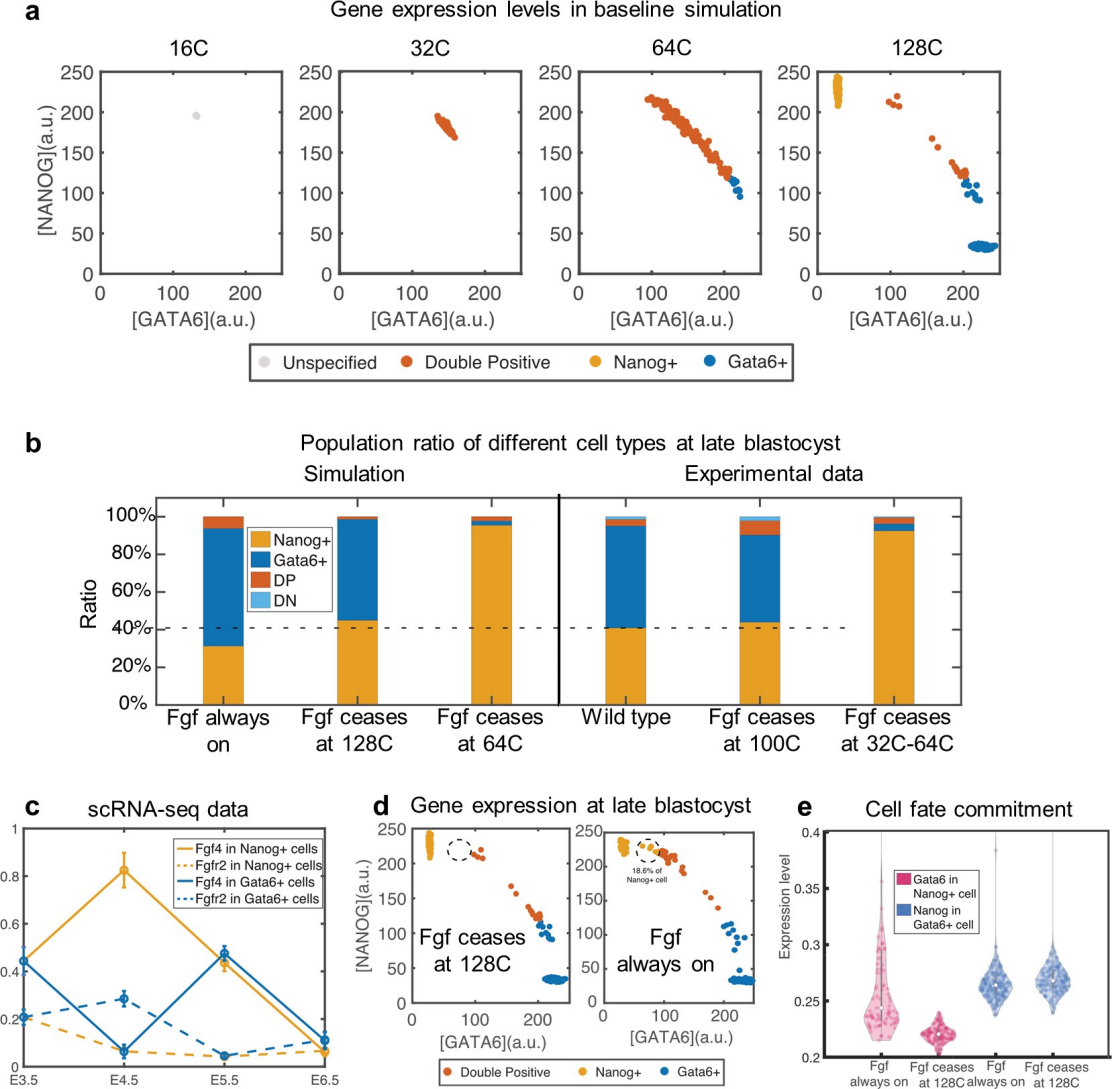
Nanog/Gata6 pattern for different Fgf4 ceasing time. (a) Single cell gene expression level in different cell stages in baseline simulation. (b) Cell number percentage of DP/Nanog+/Gata6+/DN cells at different 128C (simulation)/ 100C-150C (experiment) stages with different Fgf ceasing time in simulations and experiments. The horizontal dashed line shows the percentage of Nanog+ cells in wild type experiment. (c) Fgf4/Fgfr2 expression levels (log1p transformed) in Nanog+/Gata6+ cells in different cell stages from scRNA-seq data. The vertical bar shows the standard deviation. T-tests were used to compare between Nanog+ and Gata6+ cells the Fgf4 or Fgfr2 expression levels from E4.5 to E6.5. The (t-statistic, two-tailed p-value) for Fgf4 at E4.5, E5.5, and E6.5 are (11.75, <1e-16), (-0.80, 0.43), and (-1.29, 0.20), respectively; and for Fgfr2 at E4.5, E5.5, and E6.5 are (-3.83, 2.2e-4), (-0.27, 0.78), and (-1.37, 0.17), respectively. (d) Gene expression level of Nanog and Gata6 at 128 cell stage if Fgf ceases at 128C and if Fgf is always on. (e) Violin plot of Gata6 level of Nanog+ cell (red) and Nanog level of Gata6+ cell (blue) for simulation where Fgf4 is always on and simulation where Fgf4 ceases at 128 cell stage. The hollow circle shows the median. The t-statistic and two-tailed p-value are 7.44 and 1.7e-12 between the first two columns, and -0.54 and 0.59 between the last two columns.

We thus evaluated how the time period during which Fgf signaling is active influences cell ratios while sorting is occurring. We first test whether the timing of Fgf signaling termination influences organization. We carried out simulations where Fgf signaling either 1) is attenuated at 128-cell stage or 2) persists through the simulation. The control parameter *ε*_*t*_ in Eqs (1) and (2) is used to modulate the effect of Fgf signaling mathematically (See S1 Text section 2 and 3 for details). Fgf attenuation leads to a Nanog+/Gata6+ cell type separation at the128-cell stage (Fig 4A) with cell type ratios consistent with experimental data [16] (Fig 4B). In contrast, when Fgf signaling remains persistent, there are more uncommitted cells (Fig 4D and 4E) and the Nanog+/Gata6+ ratio is inconsistent with experimental data (Fig 4B). Further results show that when Fgf signaling is persistent, 18.6% of Nanog+ cells highly express Gata6 (Fig 4D) and Gata6 expression is higher overall in these Nanog+ cells than when Fgf signaling is attenuated at the 64-cell stage (Fig 4E). Based on these results, we propose that the function of Fgf4/Fgfr2 on PE specification is likely to be attenuated at 128-cell stage.

To test this hypothesis, we quantified the Fgf4/Fgfr2 expression levels in Nanog+/Gata6+ cells at different embryo developmental stages using scRNA-seq data [30] (Figs 4C and S5). Results reveal that Fgf4/Fgfr2 expression levels are maximally different between Nanog+ and Gata6+ cells at ~E4.5. After this stage, expression levels are essentially the same in the two populations, suggesting Fgf signaling is no longer modulating Nanog or Gata6 in a cell type-dependent fashion. This is consistent with the model hypothesis that the function of Fgf signaling on PE specification should attenuate after organization is achieved. Experiments also show that the percentages of Nanog+/Gata6+ cells are similar in wild type and the mutants with Fgf/Fgfr inhibitor added at 128-cell stage [2,16,47], further indicating the loss of function of Fgf/Fgfr on PE specification after the 128-cell stage.

We next tested the scenario where Fgf signaling ceases at the 64-cell stage. The result shows there are only Nanog+ cells present at late 128-cell stage, consistent with experimental data (Fig 4B). These results suggest that Fgf signaling must become active at or prior to the 64-cell stage, but must cease functioning as final organization (late 128-cell stage) is reached. Fgf signaling early is required to ensure the salt and pepper distribution forms with proper cell ratios. However, Fgf signaling in this setting always tries to produce a salt and pepper distribution. Thus as the PE and Epi form through sorting, the Fgf signaling must be attenuated to ensure cells do not erroneously differentiate to reform that salt and pepper distribution.

**Fig 5:**
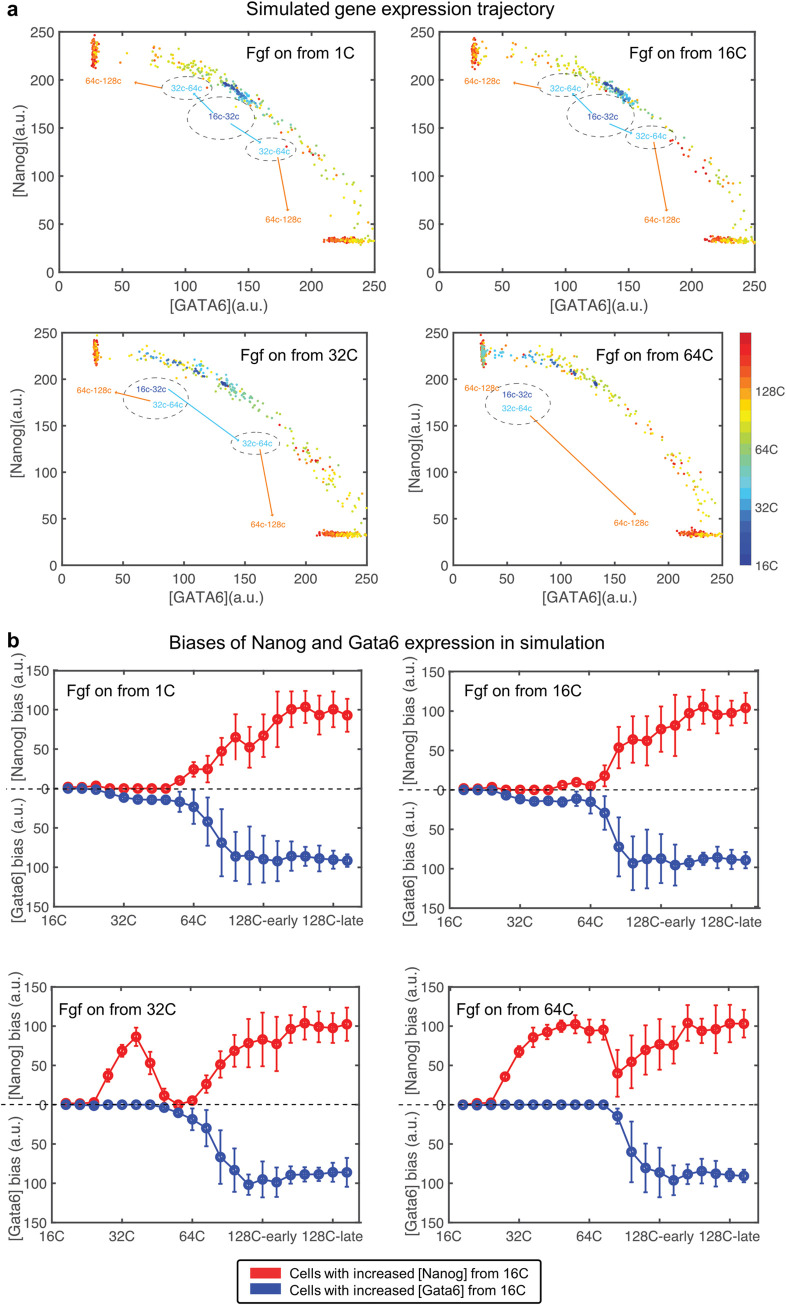
Sensitivity of Fgf on-time. (a) Trajectory of Nanog/Gata6 expression over time for different Fgf on-time. Each dot represents one cell. The color of the cell represents the stage of the cell. Dashed circles represent the range of Nanog/Gata6 expression levels for different stages: dark blue for 16C-32C, little blue for 32C-64C and orange for 64C-128C. (b) Bias of Nanog or Gata6 gene expression level from the initial state (16C) in simulation with different Fgf on-time. Red curve shows the cells whose Nanog expression is higher than cells in initial state; Blue curve shows the cells whose Gata6 expression is higher than cells in initial state. The vertical line shows the error bar with one standard deviation.

Having shown that Fgf signaling is likely to attenuate after 128-cell stage, we now investigate the beginning time of Fgf signlaing. Prior to cell fate divergence, both Nanog and Gata6 are highly expressed at the beginning of early blastocyst. This motivates us to explore whether Fgf signaling is necessary for the high expression of Nanog/Gata6 at the beginning of early blastocyst stage. We tested four scenarios where Fgf signaling begins to regulate Nanog/Gata6 expression from 1-cell, 16-cell, 32-cell, and 64-cell stages (and ceases at 128-cell stage). The expression patterns are similar between simulations with Fgf signaling beginning at 1-cell and 16-cell stages. However, if Fgf signaling does not begin until the 32-cell or 64-cell stage, cell expressions are biased to the Nanog+ state prior to Fgf onset. Even in these cases though, gene expression becomes similar to baseline simulations after Fgf signaling commences (Fig 5). This suggests that while an early starting Fgf signaling is required to form DP cells at 32–64 cell stage, a later starting Fgf signaling can still induce the formation of Nanog+/Gata6+ separation even if the system has been biased to Nanog due to the absence of early Fgf signaling. Recently, experimental studies with inhibition of Fgf signaling at different stages [2,16,47] have shown that only Epi (Nanog+) cells are present if Fgf signaling is inhibited before early blastocyst (~32-cell) stage but that PE (Gata6+) cells can be recovered if Fgf signaling is on at or after 32-cell stage. This result supports our finding that only Nanog+ cells exist when Fgf signaling is off, and some of Nanog+ cells can transfer into Gata6+ cells driven by Fgf signaling. Thus while Fgf signaling appears to be present prior to blastocyst formation, it may not be required at that stage to ensure Epi/PE formation.

Sufficiently strong gene expression noise was shown to be required to form the correct ICM/TE pattern [7]. We thus study the impact of noise levels on the expression of Nanog/Gata6 during Epi/PE formation. We considered scenarios where Nanog/Gata6 expression noise is lower (1/10 the strength) or higher (2 times the strength) than baseline levels. In the simulation, a higher noise level leads to fewer Gata6+ cells with commensurately more Nanog+ cells at 128-cell stage compared to the baseline simulation (S6 Fig). This suggests that a high noise level on Nanog/Gata6 expression can lead to an incorrect Nanog+/Gata6+ ratio.

To evaluate whether our model is able to capture correct experimental pattern in different initial conditions of Nanog/Gata6 expression, we used the Nanog/Gata6/Fgf4/Fgfr2 scRNA-seq expression data as the initial condition at 32- or 64-cell stage for simulations starting at the corresponding stages. Thus far, all initial conditions have been spontaneously determined by the gene expression ODE’s. Here, we initiated simulations from 32-cell stage and 64-cell stage (S7 Fig) using the corresponding single-cell qPCR data [29,44] as initial conditions respectively. In both simulations, correct spatial patterns formed and were similar to the imaging data at 64-cell and 128-cell stages [16] (S7 Fig). Besides, the ratio between Nanog+/Gata6+ cells was qualitatively consistent with the image data [16] when different initial conditions were used. Despite the demonstrated robustness, the model was also able to reflect the differences in initial conditions in the simulation results. A Nanog+/Gata6+ population ratio more biased to Gata6 was obtained when the initial conditions contained more Gata6+ cells (S7 Fig).

In conclusion, the model reveals that Fgf signaling is likely to attenuate after 128-cell stage, the correct Nanog+/Gata6+ population at 128-cell stage is robust to the time of Fgf signaling onset, organization can be achieved with biologically observed cell distributions of Nanog/Gata6 at the 32 and 64-cell stages, and that the noise level in Nanog/Gata6 gene expression has the potential to alter the proportion of Nanog+ and Gata6+ cells.

## Discussion

We developed a first of its kind (to our knowledge) multiscale model of development of the early embryo from the 1-cell to 128-cell stage. This three-dimensional model couples intracellular and intercellular gene regulation with selective adhesion in a realistic embryonic geometry to model the spatiotemporal development trajectory from the oocyte (1-cell stage) to blastocyst (128-cell stage). Further, we use scRNA-seq data to inform both the regulatory and the adhesion interaction parameters of this model.

Using this model, we study the roles of Fgf signaling on gene regulation, along with selective adhesion mediated cell motions on organization of the PE and Epi. This model makes two essential predictions. First, that the ligand-receptor pair EphA4/EphrinB2 or a similar mechanism is likely a major driving force behind the selective adhesion necessary to sort the initial salt and pepper distribution of cells into the PE and Epi structures. While *in vivo* embryo data is not present to confirm this finding, EphA4 and other ligands are expressed in mouse ES cells and ICM cells [45]. While models of embryonic development have been previously used to demonstrate that selective adhesion may facilitate formation of the Epi/PE, those studies had two shortcomings. First, they did not account for the presence of double-positive cells expressing both Nanog and Gata6. Second, they were phenomenological in nature and the adhesion mechanisms were not supported by experimental data. Here, we used expression of Eph/Ephrin pairs from scRNA-seq data directly parameterize our model. Results show that EphA4/EphrinB2 distributions among Nanog+ and Gata6+ cells would be sufficient to direct sorting. Thus while prior models have demonstrated the necessity of cell sorting, our results demonstrate the sufficiency specifically of EphA4/EphrinB2 in directing that sorting. This provides the first directly testable hypothesis for the presence of adhesion mediated cell sorting in the embryo.

Second, we demonstrate that attenuation of Fgf signaling is necessary after organization of the PE/Epi is achieved. Fgf signaling is known to modulate cell fate decisions during the formation of the initial salt-and-pepper distribution of cells [13–15]. We explore when this signaling should be active. Results show that proper organization is not sensitive to the timing of signaling initiation. When Fgf is not present early on, the model accurately predicts a reduced presence of DP cells commonly observed at the ~64-cell stage. However, as long as signaling is present by the 64 cell stage, cell allocations are correct as is organization. We do however find that organization strongly depends on when Fgf signaling is terminated. Fgf signaling must be attenuated after organization is achieved. Consistent with this, further single cell analysis of Fgf/Fgfr expression demonstrates that they are homogeneously distributed prior to E3.5 and after E5.5, suggesting it is not effecting gene expression in a cell type dependent fashion. Thus, it appears that Fgf may only be actively influencing gene expression during the initial formation of the salt and pepper distribution.

Both Fgf signaling and selective adhesion have been shown to be successful in regulating cell type specification and pattern formation, respectively. However, the corporation between them is mostly unknown. The model revealed that a temporal overlap of these two mechanisms could increase the robustness of early embryo development. Specifically, the model occasionally led to PE cells aggregated between TE layer and Epi cells if there is no time overlap between Fgf signaling and selective adhesion. Consistent with our model, a rare cell fate switch was shown to occur from PE to Epi, but not from Epi to PE [48]. While our model predicts that Fgf signaling is a good environmental cue mediating the process, the current evidence does not support the view [16]. However, we note that in order to address this question properly, it is necessary to track the progenitor cells over time.

The likely attenuation of Fgf signaling after the 128-cell stage raises the question, how is Fgf downregulated. We hope to explore this direction in the future by utilizing this modeling framework in conjunction with emerging data to identify potential regulators of Fgf signaling and study how they participate in later embryo developments. Currently, it is unknown how Fgf signaling is initiated such that its activity significantly increases during a short period from E3.5 to E4.5. Moreover, abundant Fgf4 expression is observed at E3.5 (Fig 4C), this 3D model can be further used to examine the recently proposed role of Fgf signaling in TE development [49]. These questions can potentially be addressed using the emerging spatial transcriptomics techniques [50–52] and integrating machine learning techniques with modeling. In general applications, modern spatial transcriptomics data will allow the construction and validation of complex models with numerous genes and interactions. This data-informed model can be built on the emerging spatial transcriptomics data and in turn, serve as a sandbox to predict the outcomes from perturbations of the mechanisms which can generate numerous *in silico* spatial data under different conditions and mechanisms.

In the temporal direction, our data-driven model can be extended to later developmental stages in future studies. This longtitudinal extension can be informed and validated with the available scRNA-seq data at later temporal points, such as the datasets up to E8.5 [32,53] or between E9.5 and E13.5 [54]. Moreover, gene regulatory networks could be extracted from scRNA-seq data in an unsupervised manner to promote the automation of the modeling framework. The potential simulated embryos beyond late blastocyst can be further validated using spatial gene expression data [55].

While this work partially relies on knowledge of gene regulatory networks and candidate regulators of selective adhesion, it is also possible to unsupervisedly integrate modeling with data to uncover novel gene-gene interactions and unknown selective adhesion genes. The data has helped us improve the model, it is worth exploring the other direction of using model to improve data analysis. For example, one can systematically screen signaling through different ligand-receptor pairs after predicting the spatial arrangements of scRNA-seq data using the model. In addition to the interactions and communications between cells, it is still an open question of how hydrodynamics in the blastocoel influences Epi/PE patterning. Since our model resembles the geometry in real biological tissues, it is well suited for the future integration of a fluid mechanics model of the blastocoel. Finally, due to the efficient implementation that harnesses GPU computing, this model could be extended to study later developmental stages involving more cells.

## Materials and methods

### Model equations and simulations

The model consists of three major components: 1) a gene regulatory network model addressing cell type specification, 2) a subcellular element model describing spatial dynamics of cells, and 3) a data-informed adhesion force model.

#### Gene regulatory network model

The gene expression dynamics of Nanog/Gata6/Fgf are represented by the following non-dimensional stochastic ODE system:

$$\frac{d{\left[Nan\right]}_{i}}{dt}=vsn_{0}+\underset{\mathrm{Inhibition}\;\mathrm{by}\;\mathrm{Erk}}{\underset{︸}{\frac{vsn_{1}\cdot Kin_{1}^{u}}{Kin_{1}^{u}+\varepsilon_{t}{\left[Erk\right]}_{i}^{u}}}}+\underset{\mathrm{Self}\;\mathrm{amplification}}{\underset{︸}{\frac{vsn_{2}{\left[Nan\right]}_{i}^{v}}{Kan^{v}+{\left[Nan\right]}_{i}^{v}}}}\cdot \underset{\mathrm{Inhibition}\;\mathrm{by}\;\mathrm{Gata}6}{\underset{︸}{\frac{Kin_{2}^{w}}{Kin_{2}^{w}+{\left[Gat\right]}_{i}^{w}}}}-\underset{\mathrm{Degradation}}{\underset{︸}{k_{N}{\left[Nan\right]}_{i}}}+\underset{\mathrm{Noise}}{\underset{︸}{\sigma_{N}{\left[Nan\right]}_{i}\cdot \eta_{N}}}$$ (1)

$$\frac{d{\left[Gat\right]}_{i}}{dt}=vsg_{0}+\underset{\mathrm{Promotion}\;\mathrm{by}\;\mathrm{Erk}}{\underset{︸}{\frac{vsg_{1}\cdot \varepsilon_{t}{\left[Erk\right]}_{i}^{r}}{Kag_{1}^{r}+\varepsilon_{t}{\left[Erk\right]}_{i}^{r}}}}+\underset{\mathrm{Self}\;\mathrm{amplification}}{\underset{︸}{\frac{vsg_{2}{\left[Gat\right]}_{i}^{s}}{Kag_{2}^{v}+{\left[Gat\right]}_{i}^{s}}}}\cdot \underset{\mathrm{Inhibition}\;\mathrm{by}\;\mathrm{Nanog}}{\underset{︸}{\frac{Kig^{q}}{Kig^{q}+{\left[Nan\right]}_{i}^{q}}}}-\underset{\mathrm{Degradation}}{\underset{︸}{k_{G}{\left[Gat\right]}_{i}}}+\underset{\mathrm{Noise}}{\underset{︸}{\sigma_{G}{\left[Gat\right]}_{i}\cdot \eta_{G}}}$$ (2)

$$\frac{d{\left[Fr\right]}_{i}}{dt}=\underset{\mathrm{Inhibition}\;\mathrm{by}\;\mathrm{Nanog}}{\underset{︸}{vsfr_{1}\frac{Kifr^{x}}{Kifr^{x}+{\left[Nan\right]}^{x}}}}+\underset{\mathrm{Prmotion}\;\mathrm{by}\;\mathrm{Gata}6}{\underset{︸}{vsfr_{2}\frac{{\left[Gat\right]}^{y}}{Kafr^{y}+{\left[Gat\right]}^{y}}}}-\underset{\mathrm{Degradation}}{\underset{︸}{k_{Fr}{\left[Fr\right]}_{i}}}+\underset{\mathrm{Noise}}{\underset{︸}{\sigma_{Fr}{\left[Fr\right]}_{i}\cdot \eta_{Fr}}}$$ (3)

$$\frac{d{\left[Fs\right]}_{i}}{dt}=\underset{\mathrm{Promotion}\;\mathrm{by}\;\mathrm{Nanog}}{\underset{︸}{vsf\frac{{\left[Nan\right]}^{z}}{Kaf^{z}+{\left[Nan\right]}_{i}^{z}}}}-\underset{\mathrm{Degradation}}{\underset{︸}{k_{Fs}{\left[Fs\right]}_{i}}}+\underset{\mathrm{Noise}}{\underset{︸}{\sigma_{Fs}{\left[Fs\right]}_{i}\cdot \eta_{Fs}}}$$ (4)

d[Erk]idt=va[Fr]i[Fp]iKd+[Fp]i⋅1−[Erk]iKa+1−[Erk]i︸PromotionbyperceivedFgf4−kErk[Erk]iKi+[Erk]i︸Degradation+σErk[Erk]i⋅ηErk︸Noise (5)

where [*Nan*]_*i*_, [*Gat*]_i_, [*Fr*]_*i*_, [*Fs*]_*i*_, and [*Erk*]_*i*_ represent secreted Nanog, Gata6, Fgfr2, Fgf4, and Erk in cell i. The perceived Fgf4 from neighboring cells for cell i is described by

$${\left[Fp\right]}_{i}=\sum_{j:|r_{i}-r_{j}|<r_{\mathrm{contact}}}(1+\gamma_{j})\frac{{\left[Fs\right]}_{j}}{N_{j}}$$ (6)

where *r*_contact_ is a cutoff determining if cells located at *r*_*i*_ and *r*_*j*_ are neighboring cells, *N*_*j*_ is the number of neighbors of cell j, and *γ*_*j*_ is a Gaussian noise.

#### Cell spatial dynamics

Every cell in the model is represented by a collection of particles (elements) in space. The movement of element j of cell i is governed by the following differential equation:

$$\frac{dr_{i,j}}{dt}=\underset{\mathrm{Intracellular}\;\mathrm{forces}}{\underset{︸}{-\nabla_{i,j}\sum_{k\neq j}V_{\mathrm{intra}}(|r_{i,j}-r_{i,k}|)}}\underset{\mathrm{Intercellular}\;\mathrm{forces}}{\underset{︸}{-\nabla_{i,j}\sum_{k\neq i}\sum_{l}\alpha \cdot V_{\mathrm{inter}}(|r_{i,j}-r_{k,l}|)}}+\underset{\mathrm{External}\;\mathrm{forces}}{\underset{︸}{F_{\mathrm{exter}}(r_{i,j})}}$$ (7)

where *r*_*i*,*j*_ is its position, α is a parameter depicting the intercelluar adhesion strengths, and *F*_exter_ is the external forces driving zona pellucida confinement and cavity formation (see S1 Text section 1 for detailed equations).

#### Connecting with scRNA-seq data

The adhesion score is estimated from the expression levels of ligand-receptor pairs in scRNA-seq data:

$$AS_{0}(CT_{i},CT_{j})={\left(\frac{{\left[L\right]}_{i}{\left[R\right]}_{j}+{\left[L\right]}_{j}{\left[R\right]}_{i}}{2}\right)}^{n_{force}}$$ (8)

$$AS(CT_{i},CT_{j})=AS_{0}(CT_{i},CT_{j})/AS_{0}(\mathrm{DP},\mathrm{DP})$$ (9)

where [*L*]_*i*_ and [*R*]_*i*_ are the average ligand and receptor expression levels among cells of type i (*CT*_*i*_) obtained from single-cell data. The relative adhesion score in Eq (9) is assigned to the parameter α in Eq (7) The parameter *n*_*force*_ corresponds to the type of adhesion modification such that *n*_*force*_ = 1 for ligand-receptor pairs that strengthen adhesion (e.g. EphA4/EphrinB2) and *n*_*force*_ = −1 for ligand-receptor pairs that weaken adhesion (e.g. EphB2/EphrinB2).

#### Embryo pattern loss score

We use a pattern loss score to quantitatively describe the successfulness of the resulting patterns in simulations. Let **x**_*i*_,1≤*i*≤*n*_icm_ be the positions of the inner cell mass at the last frame of the simulation and $t_{i}^{\mathrm{mdl}}$ be the cell type of cell i which is either Epi or PE. Assuming the embryo is centered at the origin, we assign a reference location called the ICM pole at $r\bar{x}/\left\|\bar{x}\right\|$ where $\bar{x}$ is the average position of inner cell mass and r is the average distance from TE cells to the embryo center. We then assign an ideal cell type $t_{i}^{\mathrm{ideal}}$ that best replicates the known embryo pattern by assigning the *n*_epi_ cells closest to the ICM pole as Epi type and the rest as PE type. From these two cell type assignments, we define two index collections, $J_{1}=\{i:t_{i}^{\mathrm{mdl}}=\mathrm{Epi},t_{i}^{\mathrm{ideal}}=\mathrm{PE}\}$ and $J_{2}=\{i:t_{i}^{\mathrm{mdl}}=\mathrm{PE},t_{i}^{\mathrm{ideal}}=\mathrm{Epi}\}$. The difference between these two cell type assignments is quantified by $d_{\mathrm{mdl}}=\underset{\gamma \in \Gamma }{\inf}\sum_{j\in J_{1}}\left\|{\bar{x}}_{i}-{\bar{x}}_{\gamma (i)}\right\|$ where Γ is the collection of all bijections from *J*_1_ to *J*_2_. Similarly, we also quantify the difference between randomly assigned cell types and the ideal cell type assignment, and repeat this process to derive an empirical expected difference ${\bar{d}}_{\mathrm{rand}}$. Finally, we use $d_{\mathrm{mdl}}/{\bar{d}}_{\mathrm{rand}}$ as a normalized loss score quantifying the successfulness of the formed pattern. A simulated pattern identical to the ideal cell type assignment has a loss of 0.

#### Simulation

The model was implemented in C programming language. Parallel computing was used for the movement of elements through OpenCL. The simulations were carried out on the High Performance Computing Cluster at University of California Irvine.

### Data analysis

Two different single-cell gene expression datasets were used: 1) a scRNA-seq dataset with 721 cells and 24484 genes at four temporal points from E3.5 to E6.5 [30], and 2) a single-cell qPCR dataset measuring 48 selected genes in 442 cells from the 1-cell stage up to ~64-cell stage [29]. The spatial imaging data at early and late blastocyst consists of measurement of cell type marker genes Oct4, Cdx2, Gata6, and Nanog [9,16]. See S1 Text section 4 for data processing details.

## Supporting information

S1 Text Supplementary method. (PDF)

S1 Fig Adhesion mechanisms involving TE cells and the simulation results. (PDF)

S2 Fig Adhesion mechanisms involving DP cells and the simulation results. (PDF)

S3 Fig Simulation results with different noise levels on cell movement. (PDF)

S4 Fig Simulation results with only mutual inhibition between Nanog and Gata6. (PDF)

S5 Fig Expression of cell fate regulating genes in scRNA-seq data at different stages. (PDF)

S6 Fig Simulation results with different noise levels on Nanog and Gata6 expression. (PDF)

S7 Fig Baseline hypothesis-driven model simulation results using single-cell data as initial conditions. (PDF)
