# Supplementary material for: A multiscale model via single-cell transcriptomics reveals robust patterning mechanisms during early mammalian embryo development
Source: PLoS Comput Biol. 2021 Mar 8;17(3):e1008571. doi: 10.1371/journal.pcbi.1008571 (PMC7971879; doi:10.1371/journal.pcbi.1008571)
Supplement: S1 Text — (PDF) [file pcbi.1008571.s001.pdf]

Supplementary Method

Zixuan Cang, Yangyang Wang, Qixuan Wang, Ken W.Y. Cho, William Holmes, Qing Nie

Contents

1 Spatial model 1
1.1 Subcellular element method 1
1.2 Cell division 2
1.3 External forces 2
2 Gene network model 3
2.1 Specification of TE and ICM 3
2.2 Specification of Epi and PE 3
2.3 Model parameters 4
3 Variants of baseline multiscale model 5
3.1 Controlling timing of processes 5
3.2 Selective adhesion models 5
3.3 Model interaction with data 6
4 Data processing 6
4.1 scRNAseq data 6
4.2 Single-cell qPCR data 6

1 Spatial model

1.1 Subcellular element method

Subcellular element method [1] represents a cell by a collection of elements in space. The movement of element j of cell i is governed by the following differential equation:

$$\begin{aligned}\frac{d\mathbf{r}_{i,j}}{dt} = & \underbrace{-\nabla_{i,j}\sum_{k\neq j} V_{\text{intra}}(|\mathbf{r}_{i,j}-\mathbf{r}_{i,k}|)}_{\text{Intracellular forces}} \underbrace{-\nabla_{i,j}\sum_{k\neq i}\sum_{l}\alpha\cdot V_{\text{inter}}(|\mathbf{r}_{i,j}-\mathbf{r}_{k,l}|)}_{\text{Intercellular forces}} \\ & + \underbrace{F_{\text{exter}}(\mathbf{r}_{i,j})}_{\text{External forces}} + \underbrace{F_{\text{image}}(\mathbf{r},\mathbf{g}_{\text{mdl}},\mathbf{g}_{\text{spa}})}_{\text{Image data-driven}},\end{aligned} \tag{1}$$

where $\mathbf{r}_{i,j}$ is its position, α is a parameter depicting the intercellular adhesion strengths, and F_{exter} is the external forces driving zona pellucida confinement and cavity formation. The term F_{image} is the image data-driven part using the modeled expression $\mathbf{g}_{\text{mdl}}$ and expression in imaging data $\mathbf{g}_{\text{spa}}$ of some spatial reference gene. Lennard-Jones potential commonly used to describe

atomic interactions is used here for the potential functions (V_{intra} and V_{inter}) describing interactions between elements:

$$V(r) = \varepsilon\left[\left(\frac{r_{\text{m}}}{r}\right)^{12} - 2\left(\frac{r_{\text{m}}}{r}\right)^{6}\right], \tag{2}$$

where r_{m} is the distance that minimizes the potential function.

1.2 Cell division

The simulation begins with 1280 elements representing a single cell and ends with 128 cells each represented by 10 elements. When dividing a cell into two descendants, a dividing plane of random orientation is placed where it could divide the elements into two groups of the same size. The timing of the division was scheduled as the following. There are six milestone time points when the system reaches 1, 2, 4, 8, 16, 32, 64 cells. At each milestone time point, the division time for each cell was drawn from a uniform distribution. This approach mimics the process that the cell divisions are partially synchronized where they do not divide at the same time but they likely share similar growth speed. Once a cell is divided, its gene expression is carried on by its two descendants.

1.3 External forces

The confinement of cells from zona pellucida and the formation of the inner cavity are realized by applying external forces (F_{exter} in Eq. (1)) to the elements.

Here we model the zona pellucida as a sphere with a fixed volume centered at the origin. For an element $\mathbf{r}_{i,j}$, its adhesion to the membrane is modeled by

$$F_{\text{exter-zona}}(\mathbf{r}_{i,j}) = \frac{\mathbf{r}_{i,j}}{r_{\text{embryo}}}\min\left\{F_{\max}, F_0 \big/ (r_{\text{embryo}} - |\mathbf{r}_{i,j}|)\right\}, \tag{3}$$

where r_{embryo} is the fixed radius of the embryo, $F_{\max}$ is a cutoff value to stabilize the movements, and F_0 is a coefficient for the force magnitude. In the implementation, if the position $\mathbf{r}_{i,j}$ becomes outside of the sphere after a simulation step, it is moved back into the sphere by setting its position to $(r_{\text{embryo}} - \epsilon)\dfrac{\mathbf{r}_{i,j}}{|\mathbf{r}_{i,j}|}$, where ϵ is a positive parameter with a small value.

After compaction, cavity is known to form by fluid inside the embryo. Many factors contribute to the formation of cavity such as the sophisticated salt and osmotic transport. Since our focus is not on the cavity fluids, for simplicity, we use a few phantom elements to occupy the space of cavity so that the regular elements representing cells could not enter the cavity. Specifically, when introducing the cavity, the cells should have differentiated into TE and ICM. We place the first phantom element on the zona pellucida that is farthest away from the current geometric center of ICM. Then, four more phantom elements are placed on the zona pellucida around this first one such that they are equally spaced and form a plane which is perpendicular to the direction specified by the first phantom element with a distance to the sphere center equal to 2/3 of the sphere radius. The forces due to these phantom elements are applied to elements $\mathbf{r}_{i,j}$ in ICM and are defined as

$$F_{\text{exter-cavity}}(\mathbf{r}_{i,j}) = -F_{\text{rep}} \sum_k \frac{\mathbf{r}_{i,j} - \mathbf{r}_{\text{phantom},k}}{\left|\mathbf{r}_{i,j} - \mathbf{r}_{\text{phantom},k}\right|}, \quad (4)$$

where $\mathbf{r}_{\text{phantom},k}$ is the position of the k th phantom element and F_{rep} is a coefficient for the repulsion force that pushes the ICM away from the cavity.

2 Gene network model

2.1 Specification of TE and ICM

A mutual inhibition model of Oct4/Cdx2 is used to model the specification of TE and ICM [2]. The model is implemented as ODEs:

$$\frac{d[Oct]_i}{dt} = k_O \underbrace{\left(b_O + a_O \frac{[Oct]_i^n}{\theta_O^n + [Oct]_i^n}\right)}_{\text{Self amplification}} \underbrace{\left(1 + I_{CO} \frac{\theta_C^n}{\theta_C^n + [Cdx]_i^n}\right)}_{\text{Inhibition by Cdx2}} - \underbrace{d_O [Oct]_i}_{\text{Degradation}} + \underbrace{\sigma_O [Oct]_i \cdot \eta_O}_{\text{Noise}}, \quad (5)$$

$$\frac{d[Cdx]_i}{dt} = k_C \underbrace{\left(b_C + S_i + a_C \frac{[Cdx]_i^n}{\theta_C^n + [Cdx]_i^n}\right)}_{\text{Self amplification}} \underbrace{\left(1 + I_{OC} \frac{\theta_O^n}{\theta_O^n + [Oct]_i^n}\right)}_{\text{Inhibition by Cdx2}} - \underbrace{d_C [Cdx]_i}_{\text{Degradation}} + \underbrace{\sigma_C [Cdx]_i \cdot \eta_C}_{\text{Noise}}, \quad (6)$$

where $[Oct]_i$ and $[Cdx]_i$ represent the relative expression levels of Oct4 and Cdx2 in cell i. The term η is a noise term of zero mean and unit standard deviation. In Eq. (6), S_i represents the cell contact impact on Cdx expression. The cell contact parameter S_i is defined as 1.5(number of outer elements)/(number of all elements). An element in a cell is regarded an outer element if its distance from the embryo boundary is shorter than 1. This parameter describes the contact intensity between a cell the embryo boundary.

2.2 Specification of Epi and PE

The specification of Epi and PE is modeled by a mutual inhibition model of Nanog/Gata6 mediated by Fgf signaling. The ODEs for the model are defined as:

$$\frac{d[Nan]_i}{dt} = vsn_0 + \underbrace{\frac{vsn_1 \cdot Kin_1^u}{Kin_1^u + \varepsilon_t [Erk]_i^u}}_{\text{Inhibition by Erk}} + \underbrace{\frac{vsn_2 [Nan]_i^v}{Kan^v + [Nan]_i^v}}_{\text{Self amplification}} \cdot \underbrace{\frac{Kin_2^w}{Kin_2^w + [Gat]_i^w}}_{\text{Inhibition by Gata6}} - \underbrace{k_N [Nan]_i}_{\text{Degradation}} + \underbrace{\sigma_N [Nan]_i \cdot \eta_N}_{\text{Noise}}, \quad (7)$$

$$\frac{d[Gat]_i}{dt} = vsg_0 + \underbrace{\frac{vsg_1 \cdot \varepsilon_t [Erk]_i^r}{Kag_1^r + \varepsilon_t [Erk]_i^r}}_{\text{Promotion by Erk}} + \underbrace{\frac{vsg_2 [Gat]_i^s}{Kag_2^v + [Gat]_i^s}}_{\text{Self amplification}} \cdot \underbrace{\frac{Kig^q}{Kig^q + [Nan]_i^q}}_{\text{Inhibition by Nanog}} - \underbrace{k_G [Gat]_i}_{\text{Degradation}} + \underbrace{\sigma_G [Gat]_i \cdot \eta_G}_{\text{Noise}}, \quad (8)$$

$$\frac{d[Fr]_i}{dt} = \underbrace{vsfr_1 \frac{Kifr^x}{Kifr^x + [Nan]^x}}_{\text{Inhibition by Nanog}} + \underbrace{vsfr_2 \frac{[Gat]^y}{Kafr^y + [Gat]^y}}_{\text{Prmotion by Gata6}} - \underbrace{k_{Fr} [Fr]_i}_{\text{Degradation}} + \underbrace{\sigma_{Fr} [Fr]_i \cdot \eta_{Fr}}_{\text{Noise}}, \quad (9)$$

$$\frac{d[Fs]_i}{dt} = \underbrace{vsf \frac{[Nan]^z}{Kaf^z + [Nan]_i^z}}_{\text{Promotion by Nanog}} - \underbrace{k_{Fs} [Fs]_i}_{\text{Degradation}} + \underbrace{\sigma_{Fs} [Fs]_i \cdot \eta_{Fs}}_{\text{Noise}}, \quad (10)$$

$$\frac{d[Erk]_i}{dt} = \underbrace{va[Fr]_i \frac{[Fp]_i}{Kd+[Fp]_i} \cdot \frac{1-[Erk]_i}{Ka+1-[Erk]_i}}_{\text{Promotion by perceived Fgf4}} - \underbrace{k_{Erk} \frac{[Erk]_i}{Ki+[Erk]_i}}_{\text{Degradation}} + \underbrace{\sigma_{Erk}[Erk]_i \cdot \eta_{Erk}}_{\text{Noise}}, \quad (11)$$

where $[Nan]_i$, $[Gat]_i$, $[Fr]_i$, $[Fs]_i$, and $[Erk]_i$ represent secreted Nanog, Gata6, Fgfr2, Fgf4, and Erk in cell i. The perceived Fgf4 from neighboring cells for cell i is described by

$$[Fp]_i = \sum_{j:|\mathbf{r}_i - \mathbf{r}_j| < r_{contact}} (1+\gamma_j) \frac{[Fs]_j}{N_j}, \quad (12)$$

where $r_{contact}$ is a cutoff determining if cells located at $\mathbf{r}_i$ and $\mathbf{r}_j$ are neighboring cells N_j is the number of neighbors of cell j, and γ_j is a Gaussian noise. The cutoff distance for cell-cell contact is defined as $r_{\text{contact}} = \frac{\sqrt{3}}{2}(r_i + r_j)$, where r_i and r_j are the average radius of the cell types of cell i and j.

2.3 Model parameters

The following parameters are used for the baseline simulations of the hypothesis-driven multiscale model.

Table S1 Parameters in Eq. (5) and Eq. (6). The parameters for the dimensionless equations are taken from ref. [2].

Parm.	Value	Parm.	Value	Parm.	Value
k_O	0.32	θ_O	0.5	σ_O	1.0
k_C	0.32	θ_C	0.5	σ_C	1.0
b_O	2.0	I_{CO}	1.5	η_O	$\sim \mathcal{N}(0,1)$
b_C	0.7	I_{OC}	1.5	η_C	$\sim \mathcal{N}(0,1)$
a_O	1.0	d_O	0.4	n	4
a_C	1.0	d_C	0.4		

Table S2 Parameters in Eq. (8-13). The parameters marked with * are calibrated and other parameter values are taken from ref. [3].

Parm.	Value	Parm.	Value	Parm.	Value
* vsn_0	1.1041	Kag_2	0.55	* σ_{Fs}	1.0
vsn_1	0.0051	* σ_G	1.0	va	20.0
Kin_1	0.28	k_G	0.2	Kd	2.0
vsn_2	0.321	q	4	Ka	0.7
Kan	0.55	r	3	Ki	
* σ_N	1.0	s	4	k_{Erk}	3.3
k_N	0.2	Kig	1.6	* σ_{Erk}	1.0

u	3	$vsfr_1$	2.8	x	1
v	4	$Kifr$	0.5	y	1
w	4	$vsfr_2$	2.8	z	4
ϵ_t	$\begin{cases} 1, \text{if } t_0 \le t < t_1 \\ 0, \text{otherwise} \end{cases}$	$Kafr$	0.5	$*\eta_N$	$\sim \mathcal{N}(0,1)$
Kin_2	1.604	k_{Fr}	1.0	$*\eta_G$	$\sim \mathcal{N}(0,1)$
$*vsg_0$	0.04103	$*\sigma_{Fr}$	1.0	$*\eta_{Fr}$	$\sim \mathcal{N}(0,1)$
vsg_1	0.04032	vsf	0.6	$*\eta_{Fs}$	$\sim \mathcal{N}(0,1)$
Kag_1	0.28	Kaf	5.0	$*\eta_{Erk}$	$\sim \mathcal{N}(0,1)$
vsg_2	0.321	k_{Fs}	0.077	γ_j	$\sim \mathcal{N}(0,1)$
Ki	0.7				

3 Variants of baseline multiscale model

We describe how the different timing of the processes and the selective adhesion mechanisms are implemented in the hypothesis-driven model.

3.1 Controlling timing of processes

The simulation is carried out numerically with 260000 temporal steps. The embryo reaches 2-cell, 4-cell, 8-cell, 16-cell, 32-cell, 64-cell, 128-cell states at temporal steps 10000, 20000, 30000, 40000, 50000, ~95000, and ~120000 respectively. In different *in silico* experiments, we set the on or off time of FGF signaling or selective adhesion to 16-cell, 32-cell, 64-cell, and 128-cell stages by beginning or terminating the processes at steps 39000, 75000, 95000, and 120000 respectively. These simulation steps correspond to the t_0 and t_1 values in Table S2 that controls the value of ϵ_t.

3.2 Selective adhesion models

The potential function governing the intercellular interactions will generate repulsion forces if the cells are too close or attraction forces otherwise. We implement different cell-type dependent selective adhesion models by multiplying the generated forces with coefficients depending on the force direction.

Table S3 Parameters for selective adhesion models. H1: no SA; H2: Symmetric SA; H3: Non-biased asymmetric SA; H4: Asymmetric SA with DP behavior biased to Gata6+; H5: Asymmetric SA with DP behavior biased to Nanog+; H6: EphrinB2/EphA4 induced SA; H7: EphrinB2/EphB2 induced SA.

	H1		H2		H3		H4		H5		H6		H7	
	Rep.	Attr.	Rep.	Attr.	Rep.	Attr.	Rep.	Attr.	Rep.	Attr.	Rep.	Attr.	Rep.	Attr.
Epi-Epi	0.25	4	0.25	4	0.25	4	0.25	4	0.25	4	0.266	3.756	1.19	0.84
PE-PE	0.25	4	0.25	4	1	0.75	1	0.75	1	0.75	0.850	1.176	0.765	1.308
TE-TE	1	1	1	1	1	1	1	1	1	1	1	1	1	1
DP-DP	0.25	4	0.25	4	0.25	4	0.25	4	1	0.75	0.25	4	0.25	4
Epi-PE	0.25	4	2	1	2	1	2	1	2	1	0.474	2.108	0.614	1.628
Epi-TE	1	1	1	1	1	1	1	1	1	1	1	3	1	3
Epi-DP	0.25	4	0.25	4	0.25	4	0.25	4	2	1	0.209	4.788	0.538	1.86

PE-TE	1	1	1	1	6	0.1	6	0.1	6	0.1	6	0.1	6	0.1
PE-DP	0.25	4	2	1	0.25	4	2	1	1	0.75	0.384	2.6	0.304	3.288
TE-DP	1	1	1	1	1	1	1	1	6	0.1	1	1	1	1

3.3 Model interaction with data

Data informed components in models:

1) In models H6 and H7, the parameters of the selective adhesion mechanism are inferred using scRNA-seq data [4] based on the expression of Nanog, Gata6, EphA4, EphB2, EphrinB2.
2) In the simulation in S7 Figure, the scRNA-seq data [4] and single-cell qPCR data [5] (Nanog/Gata6 expression) were used as initial conditions for the models.

Validation of the model simulations:

1) The spatial pattern and cell type composition at late blastocyst are validated by the 3-dimensional imaging data [6].
2) The observed attenuation of Fgf signaling activity from the model was supported by the analysis of Fgf expression levels in scRNA-seq data [4].
3) The single-cell qPCR data [5] is used to validate the early stage of simulations (1C to 32C stages).

4 Data processing

4.1 scRNAseq data

The original data [4] was downloaded using the GEO accession code GSE100597. The count matrix was preprocessed with log1p operation. The cell types (Nanog+, Gata6+, double positive, or double negative) were determined based on the expression levels of Nanog/Gata6.

4.2 Single-cell qPCR data

The single-cell qPCR data [5] was downloaded as supplementary data of the original publication. We followed the original publication to assign cell types for 32C and 64C stage cells. A PCA was first done on the set of 64C cells. We then used k-means clustering with k=3 to split the cells into three clusters. The marker gene expressions were used to assign the cell types (TE: Cdx2, Epi: Nanog, PE: Gata6). The 32C cells were projected to the PCA space of 64C cells. The cells were separated into two clusters using k-means clustering with k=2. The marker gene expressions were then used to assign the cell types to the clusters (TE: Cdx2, ICM: Oct4).

References

1. Newman TJ. Modeling Multicellular Systems Using Subcellular Elements. Mathematical Biosciences and Engineering. 2005;2(3):611-22.
2. Holmes WR, de Mochel NSR, Wang Q, Du H, Peng T, Chiang M, et al. Gene expression noise enhances robust organization of the early mammalian blastocyst. Plos Comput Biol. 2017;13(1):e1005320.
3. Bessonnard S, De Mot L, Gonze D, Barriol M, Dennis C, Goldbeter A, et al. Gata6, Nanog and Erk signaling control cell fate in the inner cell mass through a tristable regulatory network. Development. 2014;141(19):3637-48.

4. Mohammed H, Hernando-Herraez I, Savino A, Scialdone A, Macaulay I, Mulas C, et al. Single-Cell Landscape of Transcriptional Heterogeneity and Cell Fate Decisions during Mouse Early Gastrulation. Cell Reports. 2017;20(5):1215-28. doi: 10.1016/j.celrep.2017.07.009.
5. Guo G, Huss M, Tong GQ, Wang C, Sun LL, Clarke ND, et al. Resolution of cell fate decisions revealed by single-cell gene expression analysis from zygote to blastocyst. Developmental cell. 2010;18(4):675-85.
6. Saiz N, Williams KM, Seshan VE, Hadjantonakis A-K. Asynchronous fate decisions by single cells collectively ensure consistent lineage composition in the mouse blastocyst. Nature communications. 2016;7:13463.
