# Supplementary material for: A multiscale model via single-cell transcriptomics reveals robust patterning mechanisms during early mammalian embryo development
Source: PLoS Comput Biol. 2021 Mar 8;17(3):e1008571. doi: 10.1371/journal.pcbi.1008571 (PMC7971879; doi:10.1371/journal.pcbi.1008571)
Supplement: S1 Fig — (PDF) [file pcbi.1008571.s002.pdf]

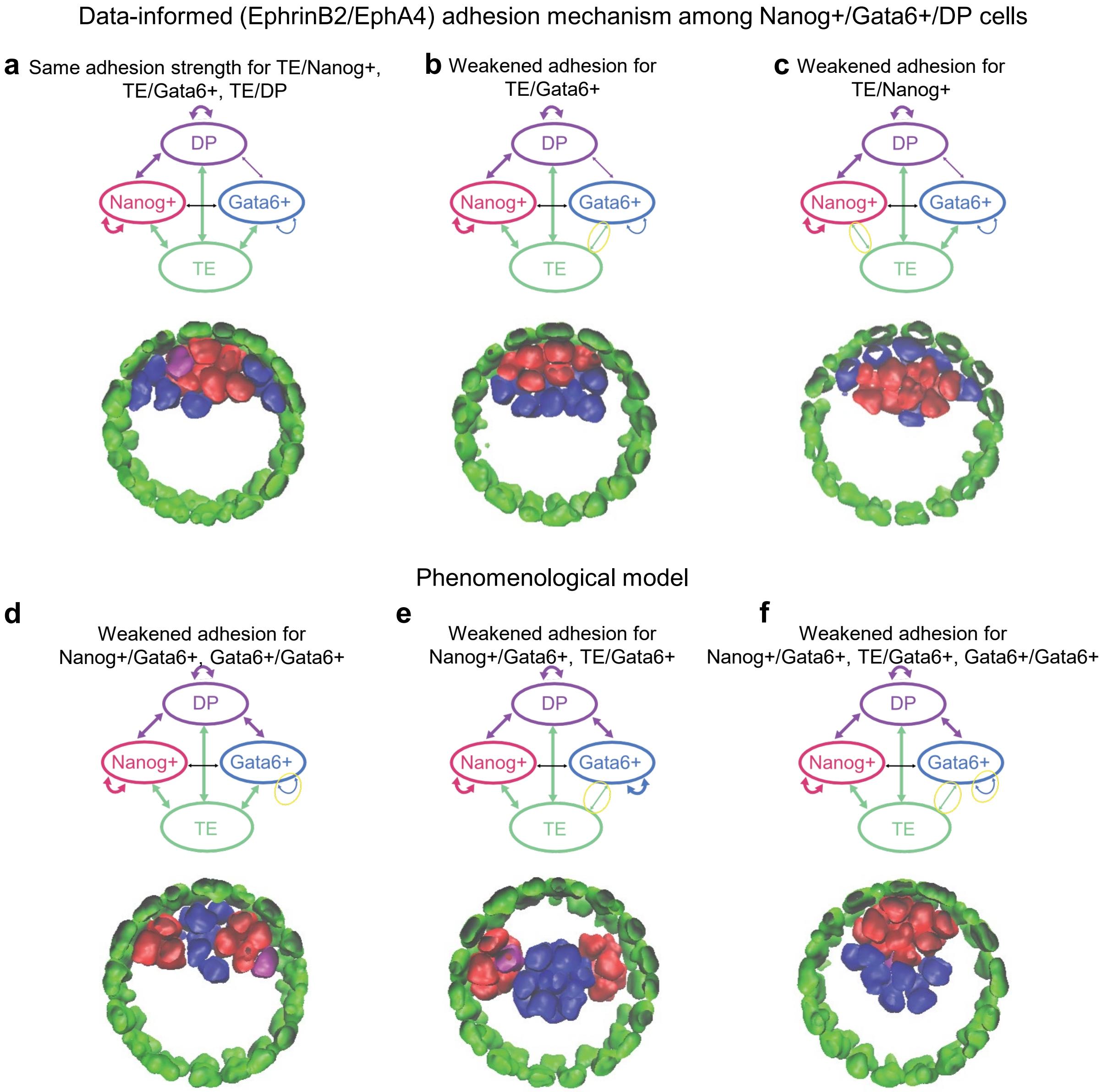

Figure S1. Adhesion mechanisms involving TE cells and the simulation results. **a-c.** Different adhesion mechanisms between TE cells and other cell types. The adhesion mechanism among Nanog+/Gata6+/DP cells is inferred from data based on the ligand-receptor pair EphrinB2/EphA4. **d-f.** Phenomenological models about selective adhesion involving TE cells.
