# Supplementary material for: A multiscale model via single-cell transcriptomics reveals robust patterning mechanisms during early mammalian embryo development
Source: PLoS Comput Biol. 2021 Mar 8;17(3):e1008571. doi: 10.1371/journal.pcbi.1008571 (PMC7971879; doi:10.1371/journal.pcbi.1008571)
Supplement: S2 Fig — (PDF) [file pcbi.1008571.s003.pdf]

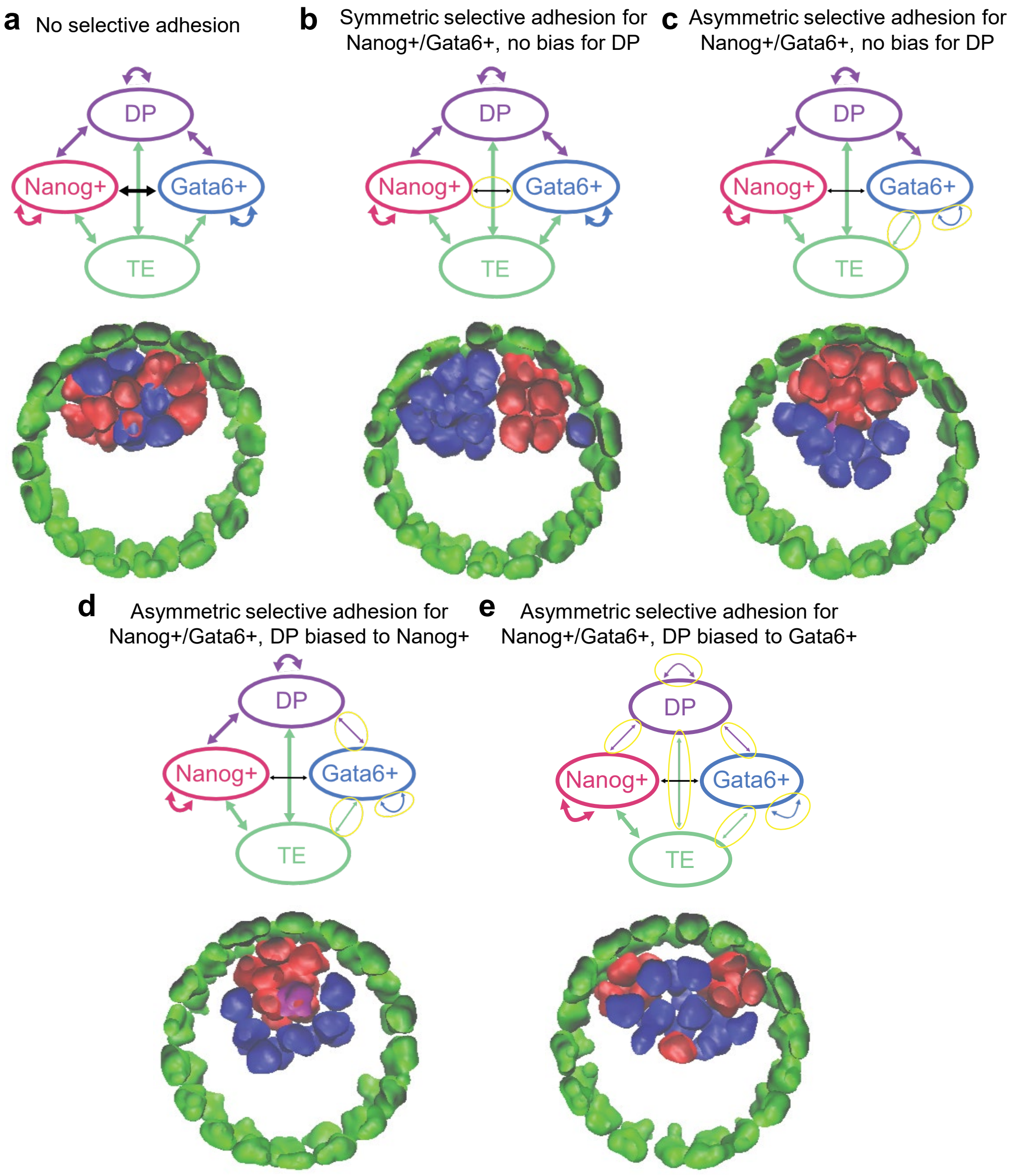

Figure S2. Adhesion mechanisms involving DP cells and the simulation results. **a-c.** Different selective adhesion mechanisms without bias for DP. **d, e.** Different selective adhesion mechanisms with biased adhesion for DP.
