# Supplementary figures and images for: A multiscale model via single-cell transcriptomics reveals robust patterning mechanisms during early mammalian embryo development
Source: PLoS Comput Biol. 2021 Mar 8;17(3):e1008571. doi: 10.1371/journal.pcbi.1008571 (PMC7971879; doi:10.1371/journal.pcbi.1008571)

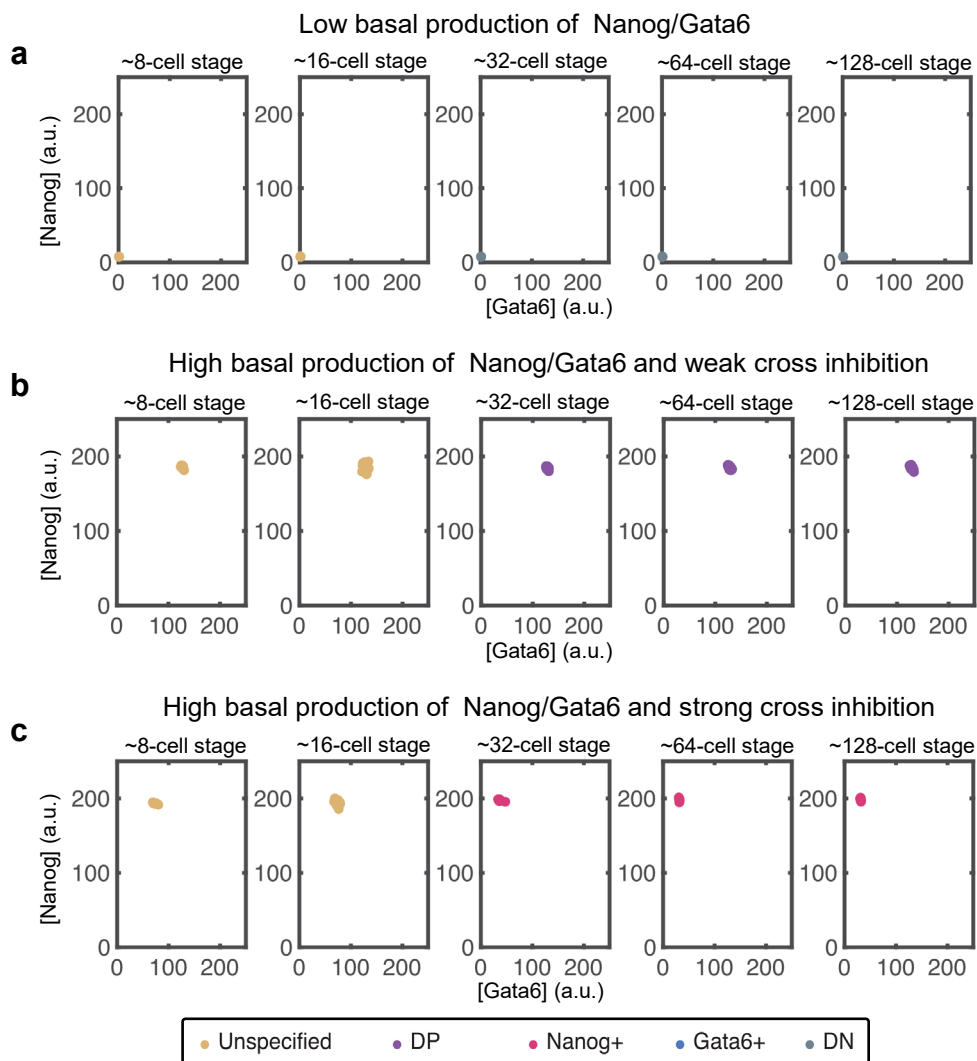

**Figure S4.** Simulation results with only mutual inhibition between Nanog and Gata6.

Supplement: S4 Fig — (PDF) [file pcbi.1008571.s005.pdf]
