# Supplementary material for: A multiscale model via single-cell transcriptomics reveals robust patterning mechanisms during early mammalian embryo development
Source: PLoS Comput Biol. 2021 Mar 8;17(3):e1008571. doi: 10.1371/journal.pcbi.1008571 (PMC7971879; doi:10.1371/journal.pcbi.1008571)
Supplement: S5 Fig — (PDF) [file pcbi.1008571.s006.pdf]

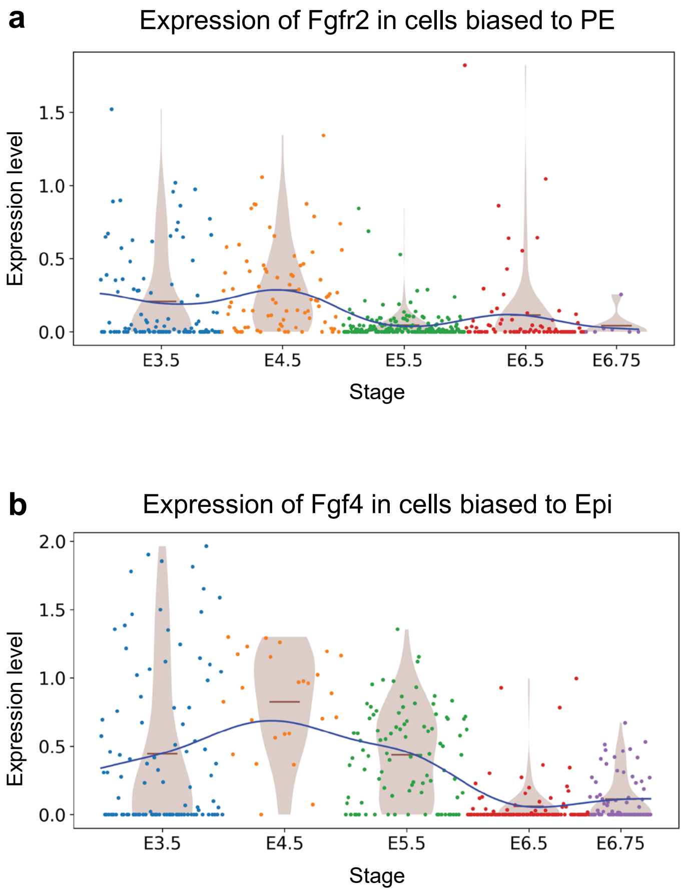

Figure S5. Expression of cell fate regulating genes in scRNA-seq data at different stages. **a.** The expression levels of Fgfr2 in cells biased to PE ([Nanog] ≤ [Gata6]) The curve is obtained from Gaussian process regression showing the trend and the bars in the violin plots show the mean values. Values after log1p transform of the original counts are used. **b.** Similar to (a) but for Fgf expression in cells biased to Epi ([Nanog] > [Gata6]).
