# Supplementary material for: A multiscale model via single-cell transcriptomics reveals robust patterning mechanisms during early mammalian embryo development
Source: PLoS Comput Biol. 2021 Mar 8;17(3):e1008571. doi: 10.1371/journal.pcbi.1008571 (PMC7971879; doi:10.1371/journal.pcbi.1008571)
Supplement: S6 Fig — (PDF) [file pcbi.1008571.s007.pdf]

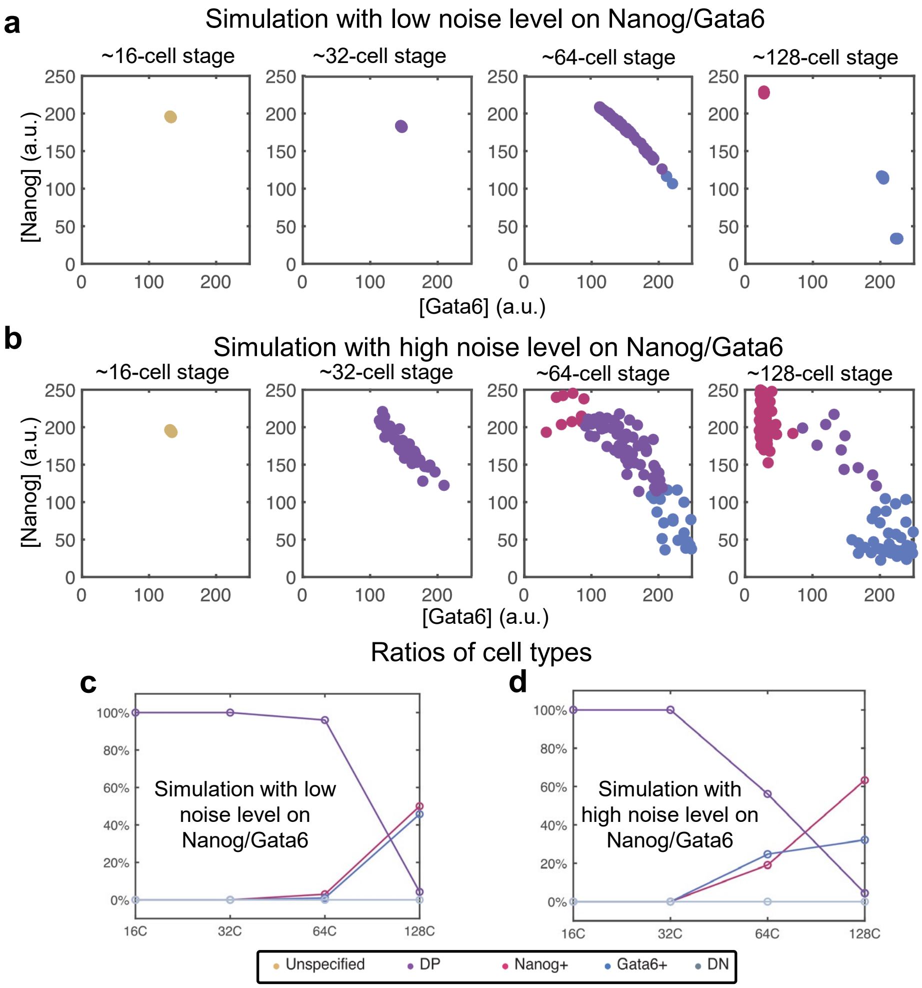

Figure S6. Simulation results with different noise levels on Nanog and Gata6 expression. **a,b.** The simulated Nanog/Gata6 expression levels with low or high noise level on Nanog and Gata6 expression. **c,d.** The ratios of cell types during simulation with low or high noise level.
