# Supplementary material for: A multiscale model via single-cell transcriptomics reveals robust patterning mechanisms during early mammalian embryo development
Source: PLoS Comput Biol. 2021 Mar 8;17(3):e1008571. doi: 10.1371/journal.pcbi.1008571 (PMC7971879; doi:10.1371/journal.pcbi.1008571)
Supplement: S7 Fig — (PDF) [file pcbi.1008571.s008.pdf]

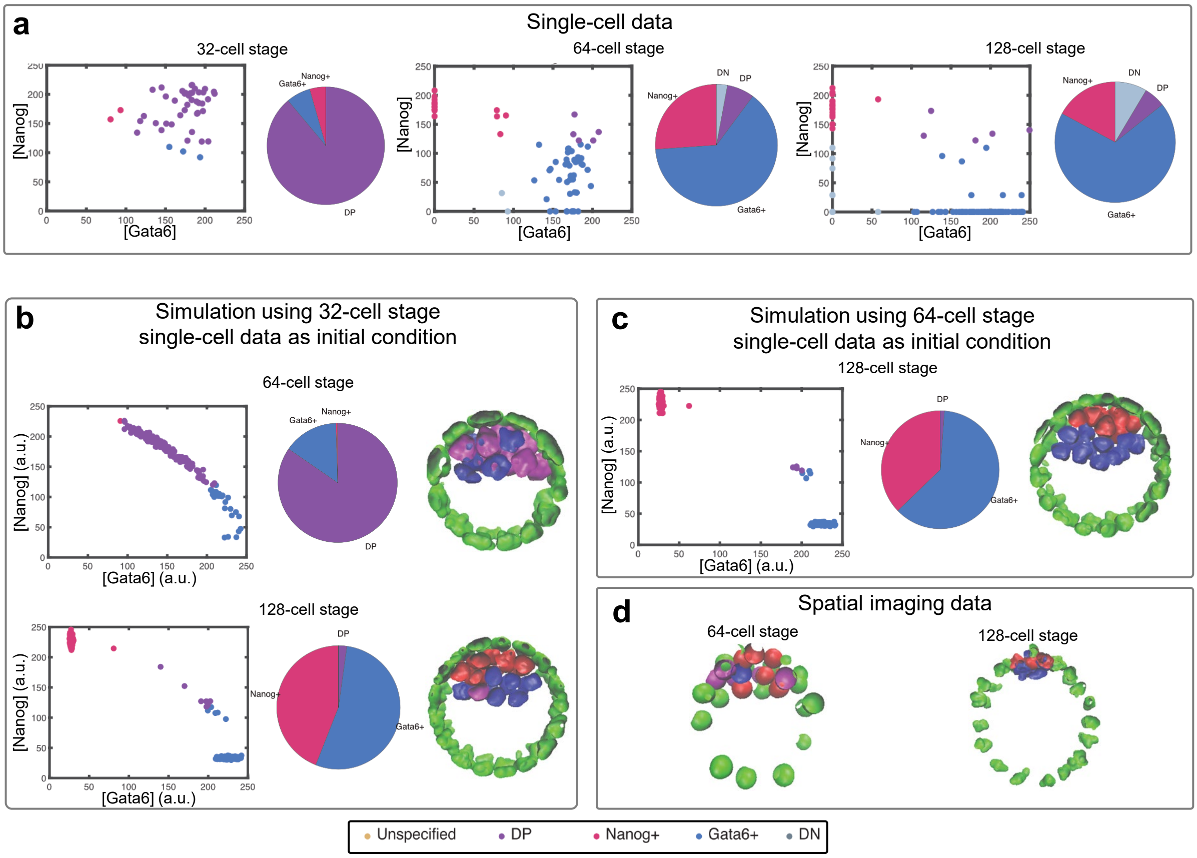

Figure S7. Baseline hypothesis-driven model simulation results using single-cell data as initial conditions. **a.** Expression of Nanog and Gata6 of ICM cells in the single-cell datasets at different stages. The 32-cell and 64-cell stage data are taken from the single-cell qPCR dataset and the 128-cell stage data is taken from the scRNA-seq data. Expression values are normalized to match the scale in the model. **b.** Simulation result of the baseline hypothesis-driven model using the 32-cell stage single-cell qPCR data as the initial condition. **c.** Simulation result of the baseline hypothesis-driven model using the 64-cell stage single-cell qPCR data as the initial condition. **d.** The spatial pattern of Nanog+ and Gata6+ cells in spatial imaging data.
